# Plant‐Pollinator Interaction Rewiring Boosts Year‐to‐Year Community Persistence

**DOI:** 10.1111/ele.70293

**Published:** 2026-01-12

**Authors:** Virginia Domínguez‐Garcia, Francisco P. Molina, Alfonso Allen‐Perkins, Oscar Godoy, Ignasi Bartomeus

**Affiliations:** ^1^ Estación Biológica de Doñana (EBD‐CSIC) Seville Spain; ^2^ Grupo de Sistemas Complejos (GSC), ETSIDI Universidad Politécnica de Madrid Madrid Spain

**Keywords:** ecological networks, interaction turnover, persistence, rewiring

## Abstract

Despite widespread recognition of the dynamic nature of ecological interactions, the consequences for community persistence of the observed year‐to‐year changes in species interactions have remained overlooked. Our research bridges this gap, leveraging a uniquely high‐quality dataset spanning 8 years and 12 independent sites—offering unparalleled resolution to examine plant‐pollinator interactions. Here, we characterise year‐to‐year variation in plant‐pollinator interactions and compare their structural stability (a robust theoretical measure describing species persistence) with that of null models simulating random rewiring. We discover that although most interaction changes (80%) are caused by species turnover, it is the temporal rewiring among permanent species that primarily enhances pollinator persistence. This interaction rewiring is not random and effectively boosts pollinator persistence despite being primarily determined by changes in the phenologies and abundances of the permanent pollinator species. While not fully optimised, these adaptive responses underscore the vital role of rewiring in fostering ecological stability amid a changing world.

## Graphical abstract

Our study shows that year‐to‐year changes in plant–pollinator interactions are not random but structured to enhance pollinator persistence, buffering communities against environmental fluctuations. Using a uniquely resolved 8‐year dataset from 12 sites, we reveal that these shifts are primarily driven by changes in phenology and abundance of the permanent species, highlighting the crucial role of rewiring in promoting ecological stability.

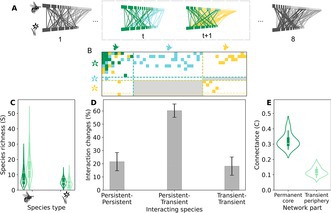

## 1 Introduction

The critical role of plant‐pollinator interactions in promoting the persistence of species within a community has been widely demonstrated, both theoretically (Bascompte et al. 2006; Bastolla et al. 2009; Rohr et al. 2014; Thébault and Fontaine 2010) and, more recently, in experimental and empirical studies (Bartomeus et al. 2021; Domínguez‐Garcia et al. 2024). However, most research on ecological networks treats these interactions as static snapshots, overlooking the dynamic nature of communities over time. There is growing recognition of the need to study the variability of the structure of ecological networks over space and time (CaraDonna et al. 2020; Chamberlain et al. 2014; Godoy et al. 2024; Poisot et al. 2014), yet this call contrasts with the few studies looking at how year‐to‐year interaction turnover affects the population dynamics of species in plant‐pollinator communities (but see (Gaiarsa et al. 2021, 2025) for two noteworthy exceptions).

What is currently known from studies evaluating the annual turnover of interactions is that plant‐pollinator communities are, in fact, extremely dynamic (Chacoff et al. 2011, 2017; Miele et al. 2020; Olesen et al. 2011; Petanidou et al. 2008; Ponisio et al. 2017; Resasco et al. 2021), with most interactions (∼70%) occurring only once throughout the studied years (Chacoff et al. 2011; Olesen et al. 2011; Petanidou et al. 2008). This temporal variability can be further described through interaction turnover, which comprises two additive components: species turnover, where interactions are lost or gained due to species entering or leaving the community; and interaction rewiring, which occurs when species that persist across years reorganise their interactions—forming new or different links among the same pool of co‐occurring species (CaraDonna et al. 2017; Poisot et al. 2012). Although this high dynamism implies that species identity is inherently variable within networks (CaraDonna et al. 2017; Costa et al. 2020; Hervías‐Parejo et al. 2023; Miele et al. 2020; Olesen et al. 2011; Ponisio et al. 2017), the general consensus is that there are conserved structures at the community level. These include the number of species, the number of interactions, the degree of nestedness—a structure in which the interactions of less connected species are subsets of those of more connected species, forming a cohesive, hierarchically organised network—and a core‐periphery structure, where a central core of highly connected generalists interacts both among themselves and with a peripheral set of less connected specialists (Chacoff et al. 2017; Miele et al. 2020; Olesen et al. 2011; Petanidou et al. 2008). However, what is yet unknown are the ecological consequences of this interaction reorganisation for the maintenance of species diversity. It has been suggested that the temporal reorganisation of plant‐pollinator networks might promote greater pollinator persistence (CaraDonna et al. 2017; Chacoff et al. 2017; Olesen et al. 2011; Petanidou et al. 2008), but this hypothesis has never been put to a critical test. An alternative scenario is that the interaction reorganisation simply reflects ecological constraints such as species abundances or phenological activity periods that limit the ability of pollinator species to interact with their preferred plant counterparts—that is, species they typically favour when co‐occurring. In fact, rewiring could also have negative consequences if it leads to increased competition for shared partners, potentially excluding weaker or more specialised species from the community. Here, we investigate the consequences for pollinator persistence of year‐to‐year changes in their mutualistic interactions with plant partners using a well‐resolved database of pollination communities over 8 years on 12 independent sites. Our hypothesis is that the observed patterns of interaction changes will promote species persistence when compared to random expectations. To test this, we compare the mutualistic networks of each site across consecutive years—examining changes in both species composition and interactions between year t and t+1. We then assess how this interaction turnover influences community persistence by applying the structuralist approach, which links species persistence to the architecture of the interaction network (Rohr et al. 2014). Specifically, we contrast the empirical patterns of interaction reorganisation with those generated by three distinct null models of interaction turnover. This framework allows us to rigorously evaluate whether interaction rewiring plays a stabilising role. Finally, we investigate whether the underlying drivers of the year‐to‐year rewiring are associated with easy‐to‐measure characteristics in the plant‐pollinator communities such as species' phenologies and abundances, that have been shown to influence interaction changes (CaraDonna et al. 2014, 2017; Resasco et al. 2021; de Manincor et al. 2020).

## 2 Materials and Methods

### 2.1 Data and Code Availability

The empirical data and codes for the study are located in Zenodo here and will be publicly available upon publication acceptance.

### 2.2 Empirical Data

Data on species population and interaction strength has been gathered during empirical observations through eight consecutive years (2015–2022) across 12 different sites situated in a landscape fragmentation gradient in the southwest of the Iberian peninsula (Huelva and Seville). Each year we sampled twice a month for at least seven rounds during the full flowering season (from February to June). The time of the day for each site was randomised across rounds, and sampling occurred only in favourable conditions (i.e., $T>18^{o}C$), at least partially sunny and without strong wind conditions. In addition, sampling was conducted from 10:00 to 17:00, overlapping with the pollinator peak activity period. We collected data on plant‐pollinator interaction frequency and flower abundance. The sites, composed by open forests islands were dominated by shrubs of the families Lamiaceae and Cistaceae, and where at least 3 km apart (mean 7.17 ± 0.97 km, Figure S1) which is larger than the foraging distances of most pollinators (Kendall et al. 2022) and hence can be viewed as independent. In each site, and during each round, we walked a 100 m straight line for 30 min in which we wrote down every plant‐pollinator interaction seen. This resulted in a total of over 330 h of field observations. Bees and plants that we were unable to identify in situ were collected for later identification. The time spent collecting specimens was discounted from the standardised sampling time. Bees were caught by hand‐netting and preserved in a freezer at −20°C until they could be pinned and labelled in the lab, and plants were preserved using plant presses. Later F.P.M. identified them using a binocular loupe and determination keys. Those specimens whose identification was unclear were sent to taxonomic experts. While non‐collected pollinators might be double counted in transects, we believe this is unlikely given the behaviour of solitary bees, and in any case, this will not affect our main analysis based on unweighted metrics. This intensive fieldwork resulted in a dataset containing 290 pollinator species and 1270 unique plant‐pollinator interactions (see Table S1). As the sampling methodology requires a different number of rounds each year to fully cover the flowering season, we verified that the effect of uneven sampling effort through the years did not affected our results by repeating the analysis in networks where the most rare species (i.e., singletons, species that appear only once each year) were removed from the community (Figures S10–S12). In addition, we assessed annual sample coverage to evaluate the proper characterisation of the interaction networks. Coverage values were generally high and consistent across sites and years, with most site–year combinations exceeding 0.7 (see Table S2). We performed this analysis using the iNEXT package (Hsieh et al. 2016).

#### 2.2.1 Annual and Potential Interaction Networks

For each site, we constructed the empirical annual interaction networks by recording all plant–pollinator interactions observed during that year, resulting in a total of eight annual empirical networks per site. We also built a corresponding set of potential interaction networks to serve as a reference for interaction opportunities. To do this, we aggregated all interactions observed across years at each site into a comprehensive meta‐network and then filtered it by retaining only those interactions involving species present in each community in a given year. The resulting potential networks represent all interactions that are both ecologically possible (species co‐occur) and plausible (the pair has interacted at least once at that site), yielding eight annual potential networks per site. We used both of these empirical networks to provide the dynamical model of mutualism (see Section 2.4 below) with a realistic backbone of interactions.

### 2.3 Quantifying Interaction Annual Reorganisation

To quantify how interactions change from year to year, we compared networks by pairs (i.e., network from year t with network from year t+1). We focused on adjacent years because interaction turnover is lower between consecutive years, indicating a degree of temporal continuity in the community (Figure S2). This approach also ensures greater species overlap across comparisons, allowing for more reliable estimates of species turnover. Looking at the networks from year t+1 we computed: (i) the number of persistent interactions that are present in both years (PI, persistent interactions), (ii) the number of gained interactions within the permanent species (GI, gained interactions because of rewiring), and (iii) the number of gained interactions as a consequence of gained species ($GI_{T}$, gained interactions because of species turnover). For those interactions gained as a consequence of species turnover (i.e., because of species that appear in year t+1 that are not present in year t), we quantified how many of those were between two new species entering the community ($GI_{T}^{N}$), or between one permanent species and a new species ($GI_{T}^{P}$). Note that $GI_{T}=GI_{T}^{N}+GI_{T}^{P}$. We then obtained the proportion of interaction changes according to the permanent/transient nature of the interaction partners (represented in Figure 2D) by dividing these quantities by the total number of interaction changes ($\mathrm{GI}+GI_{T}$). This asymmetric quantification (focused on the network at year t+1) better reflects our interest in understanding how the community observed in t+1 is assembled—whether through rewiring among persistent species or the incorporation of new species. As this framework requires information from both year t and t+1 to identify persistent species and interactions, we restricted our analysis to the 84 networks corresponding to years 2016–2022 across 12 sites—those for which data from the previous year (t) were available. While this approach differs from the symmetric quantification of previous studies (CaraDonna et al. 2017; Poisot et al. 2012), we replicated the symmetric approach and found that both frameworks yield equivalent results (Figure S13). In fact, note that the building blocks are the same as those needed to calculate interaction beta‐diversity (see Figure S3), but we prefer to analyse them separately because they are easier to understand than compound indexes (Poisot et al. 2012; Poisot 2022) and provide a more direct quantification of the contribution of rewiring and species turnover to interaction turnover (Figure S4).

### 2.4 The Mutualistic Model

To quantify the average expected persistence of pollinators (ω) following the structuralist framework, we first need to parameterize a model that describes reasonably well the population dynamics of plants and pollinators in terms of their biotic interactions. We employed a standard mutualistic dynamics model (Bastolla et al. 2009; Rohr et al. 2014; Saavedra et al. 2016; Song et al. 2018), using the empirical networks as their skeleton (see below). The equations describing the abundance of plants ($P_{i}$) and animal ($A_{i}$) species are of the form:

(1) $$\left[\begin{array}{c} \frac{\mathrm{dP}}{\mathrm{dt}}\\ \frac{\mathrm{dA}}{\mathrm{dt}} \end{array}\right]=\mathrm{diag}\left(\left[\begin{array}{c} P\\ A \end{array}\right]\right)\times \left(\left[\begin{array}{c} r_{P}\\ r_{A} \end{array}\right]-\underset{A}{\underbrace{\left[\begin{array}{cc} \alpha_{P} & -\gamma_{P}\\ -\gamma_{A} & \alpha_{A} \end{array}\right]}}\left[\begin{array}{c} P\\ A \end{array}\right]\right)$$

with parameters accounting for intrinsic growth rate (r), intraguild competition (α) and mutualistic benefit (γ). We considered mean field intra‐guild competition ($\alpha_{\mathrm{ii}}^{P}=\alpha_{\mathrm{ii}}^{A}=1$, $\alpha_{\mathrm{ij}}^{P}=\alpha_{\mathrm{ij}}^{A}=\alpha$), and used the empirical networks to parametrize the mutualistic benefit as follows: $\gamma_{\mathrm{ij}}=\left(\gamma_{0}M_{\mathrm{ij}}\right)/\left(k_{i}^{\delta }\right)$, where $M_{\mathrm{ij}}=1$ if we recorded a plant‐pollinator interaction between species i and j in the field and zero otherwise, $\gamma_{0}$ represents the overall level of mutualistic strength (i.e., the average benefit a species receives from having a mutualistic partner), and δ the mutualistic trade‐off (i.e., the extent to which species with fewer partners receive stronger per‐interaction benefits). To build a dynamical model as parsimonious as possible, we only used unweighted interactions (i.e., we considered $M_{\mathrm{ij}}$ either 1 or 0) as is usually done in this type of modelling approaches (Rohr et al. 2014; Saavedra et al. 2016; Song et al. 2018). The results in the main text correspond to parameter values *α* = 0.005, $\gamma_{0}$ = 0.1, and δ = 0, following previous studies (Saavedra et al. 2016; Song and Saavedra 2018). However, to ensure that our conclusions were robust to parameter choice, we conducted a sensitivity analysis (Table S5), confirming that our results remain consistent. While improvements can be made (e.g., considering non‐linear functional responses Cenci and Saavedra 2018), empirically based competition instead of the mean field (García‐Callejas et al. 2023), or weighted interactions, we chose this model for simplicity and because it is of standard use in previous theoretical studies and shows good predictive power (Domínguez‐Garcia et al. 2024).

### 2.5 Predicting Pollinator Persistence With the Structuralist Framework

The structuralist framework combines two critical components: species' performance and their biotic interactions. The network of biotic interactions shapes the potential for species within the community to persist (Domínguez‐Garcia et al. 2024; Godoy et al. 2018). In parallel, species' performance, defined as the set of intrinsic growth rates compatible with species persistence, reflects the species' tolerance to environmental conditions, creating a direct link between ecological dynamics and environmental factors (Song et al. 2018). Communities with larger coexistence opportunities are likely more persistent because they can tolerate larger differences in intrinsic growth rates (i.e., performance) among species. To apply this framework to pollinators, we derived the matrices describing the effective biotic interactions among pollinators ($\alpha_{A}^{\prime }$) from our mutualistic model (Equation 2) following the effective interaction methodology (Rohr et al. 2014). In this method, the mean‐field competition among insects (α, representing generalised resource competition) is modified by the mutualistic benefits they receive from interacting with plants. To obtain the effective biotic matrix ($\alpha_{A}^{\prime }$), we applied a block‐wise diagonalization of the full interaction matrix A in Equation 3. This process transforms the original system of $N_{P}+N_{A}$ coupled equations—describing the steady‐state abundances of plants and pollinators—into two decoupled systems: one for plants ($N_{P}$ species) and one for pollinators ($N_{A}$ species), each involving only intra‐guild effective interactions ($\alpha_{A}^{\prime }$ and $\alpha_{P}^{\prime }$ in Equation 3). This transformation is achieved by multiplying both left sides of Equation 2 by the matrix $T=1+\Gamma C^{-1}$ (see Supporting Information: Appendix SI1D for a more detailed explanation).

(2) $$\left[\begin{array}{c} r_{P}\\ r_{A} \end{array}\right]=\left(\overset{A}{\overbrace{\underset{C}{\underbrace{\left[\begin{array}{cc} \alpha^{P} & 0\\ 0 & \alpha_{A} \end{array}\right]}}-\underset{\Gamma }{\underbrace{\left[\begin{array}{cc} 0 & \gamma_{P}\\ \gamma_{A} & 0 \end{array}\right]}}}}\right)\left[\begin{array}{c} P\\ A \end{array}\right]$$

(3) $$\begin{array}{r} \left[\begin{array}{c} r_{P}^{\prime }\\ r_{A}^{\prime } \end{array}\right]=\left[\begin{array}{cc} \alpha_{P}^{\prime } & 0\\ 0 & \alpha_{A}^{\prime } \end{array}\right]\left[\begin{array}{c} P\\ A \end{array}\right] \end{array}$$

We quantified the expected average persistence probability of pollinators (ω) using the effective biotic interaction matrix $\alpha_{A}^{\prime }$ (Equation 3) as input to a structural stability analysis. Specifically, we used a function that calculates the proportion of intrinsic growth rate vectors (i.e., species performance combinations) that allow all species in the community to persist, given the structure of biotic interactions (code available in (Saavedra et al. 2016)). In probabilistic terms, ω reflects the likelihood that a randomly selected performance scenario permits coexistence of all species (Song et al. 2018). Higher values of ω indicate greater structural stability and expected persistence, driven solely by the mutualistic interaction network and the mutualistic model parameters.

### 2.6 Null Models of Network Reorganisation

**FIGURE 1:**
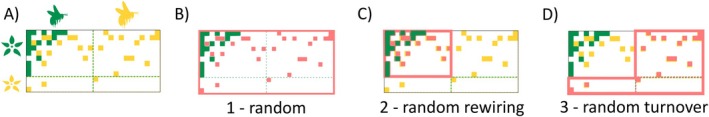
Schematic representation of the null models of annual interaction turnover: This figure illustrates how each null model reshuffles interactions while preserving key structural elements of the plant–pollinator network at year *t* + 1. (A) Empirical network of year t+1, with permanent species (i.e., those present also in year t) coloured in green, and transient species (those only present in year t+1) coloured in yellow. (B–D) illustrate the interactions that are randomised by each null‐model, coloured in red. The “random model” (B) allows reshuffling all transient interactions, the “random rewiring model” (C) allows only reshuffling transient interactions that are caused by rewiring, and the “random turnover model” (D) allows reshuffling only transient interactions that are caused by species turnover.

To determine whether the observed year‐to‐year interaction turnover in plant‐pollinator networks was organised to enhance pollinator persistence (quantified as ω) or simply reflected opportunistic attachment among the species present in a given year, we developed three null models of interaction turnover (Figure 1). These models generate synthetic versions of the networks for year t+1 such that they preserve the number of species, their permanent or transient role (i.e., whether they were present in the previous year), and the number of new interactions in year t+1 (i.e., interactions present in year t+1 that are not present in year t), but, ensuring that all the species in the randomised network have at least one mutualistic partner, they allow the transient interactions to be randomised with the following increasing constraints:

- random model: This null model reshuffles all the interactions from year t+1 with the exception of those interactions that are present both years (i.e., permanent interactions, in green in Figure 1B). It ensures that the number of interactions within each part of the network remains constant: permanent plant—permanent pollinator, permanent plant—transient pollinator, permanent pollinator—transient plant, and transient plant—transient pollinator.
- random rewiring model: This null‐model randomises the transient interactions that are a consequence of rewiring (i.e., transient interactions between permanent species, marked in red in Figure 1C).
- random turnover model: This null‐model randomises the transient interactions that are a consequence of species turnover (i.e., transient interactions involving at least one transient species, marked in red in Figure 1D), keeping constant the number of interactions in each part of the network as in the random model.

### 2.7 Comparing Empirical and Randomised Networks

For each of the 84 empirical networks (12 sites × 7 years), we generated 500 randomised counterparts using the three null models described above. These randomised networks allowed us to evaluate how annual interaction turnover influences pollinator persistence probability, quantified as ω (see Sections 2.4 and 2.5). We assessed the effect of empirical interaction structures by computing *Z*‐scores, which quantify how far the empirical ω deviates from the average of the randomised ensemble, in units of standard deviation. The calculation is as follows:

(4) $$z=\frac{x-\mu }{\sigma }$$

where x represents the specific empirical value being evaluated, μ is the mean of the distribution in the random ensemble, and σ is the standard deviation of the distribution. A positive *Z*‐score indicates that the value is above the mean, while a negative *Z*‐score signifies that it falls below the mean. Importantly, small *Z*‐scores (typically between −1 and 1) suggest that the mechanisms included in the null model are sufficient to reproduce structures similar to those observed in the empirical networks. To assess the overall performance of the empirical networks, we quantified the percentage of these 84 networks with *Z*‐scores above 1 in each of the null models. In addition, we examined the ω values of the top 1% of randomised networks—those with the highest expected pollinator persistence—as a way to identify the most optimal configurations generated by each null model and to compare their structure with that of the empirical networks (Figures S8 and S9 and Table S3 in SI2B).

### 2.8 Drivers of Rewiring

To better understand the drivers of the amount of interaction rewiring, we evaluated whether changes in species abundance and phenology, as well as interaction turnover involving transient species, could explain the observed variation in rewiring across years, as suggested by previous studies (CaraDonna et al. 2017; Resasco et al. 2021; Brosi and Briggs 2013). Note that changes in species abundances can reflect both direct effects (e.g., probability of encounter) and indirect effects (e.g., competition). For that, we built a linear mixed‐effects model of rewiring, where the independent variables were changes in the abundance of permanent pollinator and plant species, changes in the phenology of permanent pollinator and plant species, and amount of interaction turnover due to species turnover. We included the sampling site as a random effect to account for the nested structure of the dataset (12 sites sampled over 8 years). All variables were standardised prior to analysis, and models were implemented in Python using the pymer4 package (Jolly 2018). We confirmed that the assumptions of normality and homoscedasticity of residuals were met, supporting the validity of applying the linear model to the standardised data (Figure S5).

#### 2.8.1 Measuring Changes in Species' Abundances

We measured the resource abundance by recording the number of flowers of each plant species in ten 1 m^2^ subplots placed along the transect. We obtained the annual resource abundance of plant species in each site by summing the resource abundance from all rounds in each year. We measured pollinator abundance by counting the number of recorded individuals of each pollinator species. We obtained pollinators' annual abundance in each site by aggregating, through each year, pollinator abundance. In both cases, we normalised the abundances with the sampling effort of that year (i.e., the number of rounds). We then measured the total changes in the abundances of the persistent species from year t to year t+1 as:

(5) $$\Delta_{\mathrm{abn}}=\sum_{i\in p}|x_{i}\left(t+1\right)-x_{i}\left(t\right)|$$

where $x_{i}\left(t\right)$ denotes the abundance of species i at year t, $x_{i}\left(t+1\right)$ the abundance of species i at year t+1, and ‘*p*’ denotes that we take into consideration only *permanent* species, those that are present in both years.

#### 2.8.2 Measuring Changes in Species' Phenology

We quantified changes in phenology by comparing the number of days each species had been present in the field as:

(6) $$\Delta_{\text{pheno}}=\sum_{i\in p}|L_{i}\left(t+1\right)-L_{i}\left(t\right)|$$

where $L_{i}\left(t\right)$ denotes the number of days that each species was present in the field (i.e., the number of days elapsed between the first and last date the species was recorded at a given site) in year t, $L_{i}\left(t+1\right)$ the number of days each species was present in the field in year t+1, and ‘*p*’ denotes that we take into consideration only *permanent* species.

## 3 Results and Discussion

### 3.1 Large Year‐to‐Year Reorganisations Promoted by Species Turnover

**FIGURE 2:**
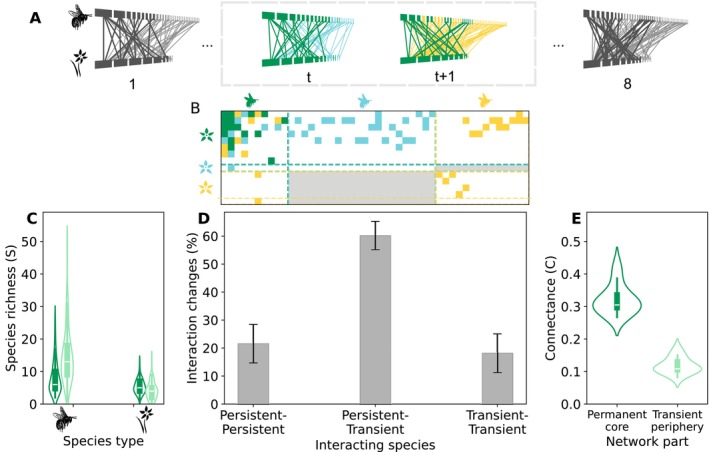
Quantification and characterisation of year‐to‐year interaction turnover in plant pollinator networks, revealing the structural importance of permanent species and the dominant role of transient species. (A) Annual plant‐pollinator networks of one of the study sites (Aznalcazar) through time, from year 1–8. We compare networks of consecutive years (t and t+1) and determine species present in both years (i.e., ‘permanent species’, in deep green) and species present only in the first or second year (‘transient species’, in light blue or yellow, respectively). We also determine which interactions are present in both years (links in deep green) and which interactions are present only in the first or second year (links in light blue and yellow, respectively). Dark and light grey lines represent, respectively, permanent and transient interactions observed outside the focal time window (*t* to *t* + 1). Node size is proportional to the number of mutualistic partners of the species. (B) The matrix representation of plant‐pollinator networks of year t and t+1. The dashed lines are a help to identify the different species sets (permanent species, and transient species from year t or t+1, respectively). The colour code for species and interactions is the same as in Figure 2A, and the grey shaded area represents impossible interactions between species that are not simultaneously present during the time window. Using these matrices, we quantified interaction changes from year t to t+1 by looking at (i) the number of persistent interactions that are present in both years (PI, persistent interactions), (ii) the number of gained interactions due to rewiring (GI), and (iii) the number of gained interactions due to species turnover ($GI_{T}$), paying attention to the permanent or transient nature of the interacting species. See Methods for further details. (C) Distribution of permanent species richness (i.e., number of species that remain for two consecutive years, in deep green) and of transient species richness (in light green) for pollinators and plants in the annual networks in our study. (D) Distribution of ecological interaction changes according to the permanent/transient nature of the interaction partners. The height of the bar represents the average value across the 84 annual networks, and the error bars represent one standard deviation. (E) Distribution of the density of interactions (i.e., connectance) in the permanent species core (formed by the permanent species) compared to its value in the transient periphery (formed by transient species).

The turnover of species is very high in our mutualistic communities, with only 5 pollinator species ( *Bombus terrestris* , *Xylocopa cantabrita* , *Dasypoda cingulata* , *Flavipanurgus venustus and Apis mellifera*), and 10 plant species (*Cistus crispus*, *Cistus ladanifer* , *Cistus monspeliensis* , *Cistus salviifolius* , *Salvia rosmarinus*, *Teucrium fruticans* , *Halimium calycinum*, *Lavandula stoechas* , *Lavandula pedunculata and Cistus libanotis*) being present for the whole duration of our study. The interactions in our plant‐pollinator communities are also highly dynamic, with most of these interactions (∼90%) being present only one or 2 years. The set of interactions recorded continuously during more than 7 years represents a minor fraction (∼1.4%). These pronounced year‐to‐year shifts are in line with previous work following plant‐pollinator communities over long periods of time (Chacoff et al. 2017; Olesen et al. 2011; Petanidou et al. 2008). To investigate these highly dynamic annual changes in interaction structure, we assessed interaction turnover by comparing networks of successive years (see Methods Section 2.3). First, we quantified the species that remain in the community across two consecutive years (hereafter ‘permanent species’, coloured in deep green in Figure 2A,B) and those that either exit or enter from 1 year to the next (hereafter ‘transient species’, coloured in light blue and yellow, respectively). Next, we quantified interactions that persisted across both years (referred to as ‘permanent interactions’, shown in deep green in Figure 2A,B) and those present in only 1 year—either the first or second (referred to as ‘transient interactions’, depicted in light blue and yellow, respectively). For transient interactions, we further examined whether they resulted from ‘rewiring’—changes between two permanent species—or from ‘species turnover’, involving either permanent‐transient pairs or two transient species (see Figure 2B).

We found that few pollinators remain present in the community during consecutive years, as shown in the violin plot representing the distributions of the number of permanent species in the annual networks (Figure 2C). On average, only ∼37% of pollinators remain, meaning that roughly two‐thirds of pollinators do not remain in the network from 1 year to the next. For plants, the situation was the reverse, with most plant species (∼58%) remaining present through two consecutive years (Figure 2C), which is expected since most are shrubs. This contrasts with what is found in fruit‐frugivore interactions, where birds were more temporally reliable than fruits (Costa et al. 2020). This may be influenced by birds being long‐lived species, which means that if a bird species is present 1 year, it probably will be present the next year too. In contrast, insects are mostly species with annual cycles which can be reflected in their increased volatility. Note, however, that part of those differences may also arise from differences in sampling, where plant‐pollinator communities are normally plant‐based while plant‐frugivores are animal‐based (Gibson et al. 2010).

We also document that most inter‐annual interaction changes are due to species turnover (∼60% between permanent and transient species and ∼20% between two transient species), while only ∼20% of the interaction changes are due to rewiring. This is in line with previous results showing that ∼70% of interaction changes were due to species turnover (Petanidou et al. 2008), but contrast with previous reports where most interaction turnover is attributed to rewiring (CaraDonna et al. 2017; Costa et al. 2020; Hervías‐Parejo et al. 2023) (although this discrepancy may be caused by the employed methodology, see Figures S3, S4 and SI1C). Despite this high variability, these communities retain a form of short‐term memory, with consecutive years being more similar to each other than those separated by longer intervals (Figure S2). It is worth mentioning that we found no significant effect of landscape composition (e.g., proportion of natural areas) on the total interaction turnover or on the proportion of rewiring (see Figure S6), suggesting that these reorganisation patterns are not strongly driven by landscape context. Interestingly, when we look at how interactions are distributed between permanent and transient species, we show that most interactions are concentrated in the core formed by the permanent species (see Figure 2E). This result supports prior evidence that generalist species tend to occupy central positions in these networks (Costa et al. 2020; Hervías‐Parejo et al. 2023; Miele et al. 2020; Resasco et al. 2021). For example, in our study system, abundant species with long flying seasons and a generalised diet, such as *Apis mellifera* , *Bombus terrestris* or *Xylocopa cantabrita* , tend to form the core of permanent species, but we also found some exceptions, such as the specialist *Flavipanurgus venustus*.

### 3.2 The Empirical Reorganisation Boosts Pollinator Persistence, but Is Not Optimal

We applied the structural stability framework to evaluate the pollinator expected persistence probability (ω, see Methods sec. 2.4 and 2.5). We observe that variation in interaction turnover across years in the 12 independent sites led to variation in the values of ω. Therefore, our results provide further evidence of the influence of the structure of the interaction network on species persistence. The magnitude of variation in the persistence probability of pollinators is moderate, with values ranging from 0.48 to 0.53. These moderate values are as expected from natural communities located within a single habitat type, where temporal weather variability is the primary environmental change (Domínguez‐Garcia et al. 2024). In other scenarios involving distinct habitats or heavily degraded environments, larger shifts in species persistence are predicted (Winfree et al. 2011).

**FIGURE 3:**
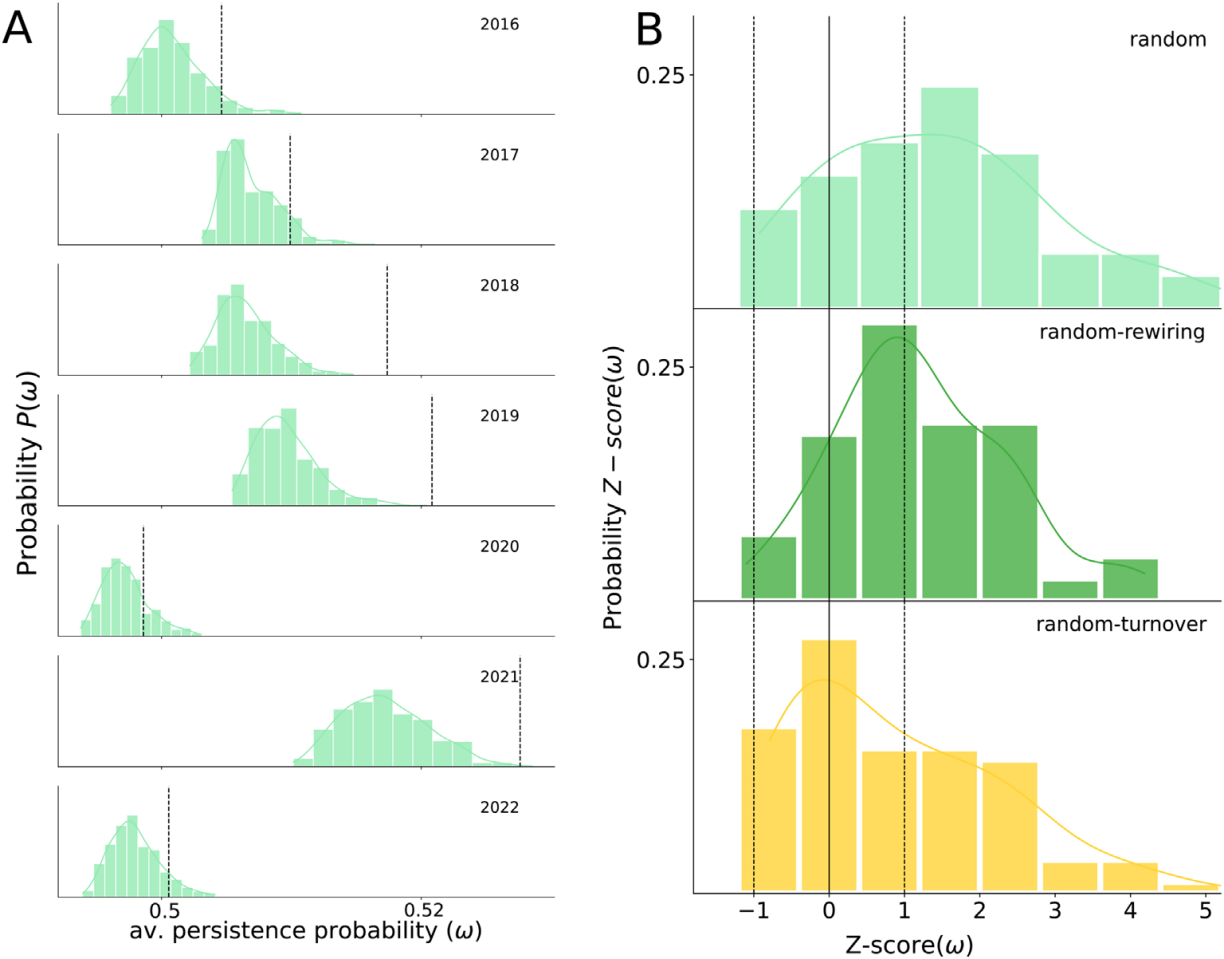
Comparison of expected pollinator persistence in empirical network and their randomised counterparts, showing that the observed empirical values are greater than what would be expected by chance alone (null models): (A) Distribution of the values of expected pollinator persistence (ω) for 500 randomised networks generated with the ‘random null model’ (the least restrictive of the studied null models), for each year in one of the study sites (‘Aznalcazar’). The black dashed line indicates the ω value of the empirical network in each year. (B) Distributions of *Z*‐scores for the average persistence probability of pollinators (ω) across the 84 empirical networks (one per site and year) under the three null models, each shown in a different colour. The solid vertical line indicates Z=0, while dashed lines mark Z=±1, the conventional thresholds for statistical significance—values beyond these suggest that the empirical network differs substantially from the average of its randomised counterparts.

Next, we evaluated the performance of the 84 empirical networks by comparing the expected pollinator persistence probability (ω) with that of their randomised counterparts (Methods Section 2.6 and 2.7). Our analysis revealed that the empirical network structures observed in the field generally enhance pollinator persistence (see Figure 3, Figure S7). Examining the annual networks from one of the study sites, we observe that the empirical values of ω (marked as dashed vertical lines) are typically higher than most of the values corresponding to the 500 randomizations in the ‘random model’ (Figure 3A). To assess this pattern across all networks, we analysed the distribution of the 84 *Z*‐scores of ω in the three null models (Figure 3B). For both the ‘random’ and ‘random‐rewiring’ null models, *Z*‐scores peak around 1, with a majority of cases (∼58%) exceeding this threshold. In contrast, in the case of the random‐turnover’ model where only the transient interactions are randomised‐ most networks (∼57%) display a *Z*‐score below one, indicating that the empirical networks are not significantly different from these randomizations. Hence, this result suggests that the higher persistence in the observed pollinator networks is largely driven by permanent species forming the permanent core, which consistently reorganise interactions in ways that boost pollinator survival (Figure 3B), despite exhibiting limited rewiring overall (Figure 2D).

Interestingly, the rewiring of many permanent plant species may be related to their generalist flower structures, such as the bowl‐shaped flowers of Cistaceas (note that 6 species of Cistaceas are included in the set of the 10 species recorded every year). In contrast, transient species contribute little to the overall persistence of the pollinator community. In particular, their interaction reorganisation mostly resembles random patterns, as suggested by the random‐turnover model (Figure 3B). Our results indicate that the process of interaction turnover, specially rewiring, promotes greater pollinator persistence than expected by chance. However, we also show that the process is not fully optimised, as persistence values are not maximal on the observed networks. Across all sites, there are other possible reorganisations emerging from the null models that render higher pollinator persistence (Figure 3B, Figure S7), an effect which is mostly determined by the number of shared mutualistic partners among species (see Figure S9, Table S3). This suggests that these unobserved interaction patterns might not occur due to constraints in which links cannot be established among species each year (e.g., due to phenology mismatches, Figure S8), and therefore, in the overall reorganisation of the plant‐pollinator networks. Notably, the potential network—which includes all interactions ever observed between the species co‐occurring in a given year—exhibits significantly higher persistence than the realised empirical networks (see Figure S7).

### 3.3 Drivers of Interaction Rewiring

**FIGURE 4:**
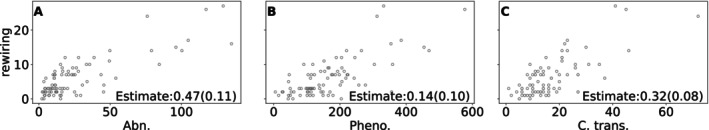
Drivers of interaction rewiring, showing that rewiring increases with changes in abundance and phenology of permanent pollinators. The figure represents the number of interaction changes caused by rewiring versus the three explanatory variables retained in the model: (A) changes in abundance of permanent pollinator species, (B) amount of phenological changes in the permanent pollinator species, and (C) number of interaction changes caused by species turnover. Each point represents one of the 84 networks in the study. Values in each panel indicate the model estimate and its standard error (in parentheses), obtained from the generalised linear mixed‐effects model in Table 1.

Given the critical role of rewiring among permanent species in maintaining the stability of plant‐pollinator communities shown above, we delved deeper into these interaction changes. To better understand which may be the underlying drivers of the observed amount of interaction rewiring, we explored to what extent the observed interaction rewiring is due to either changes in species abundances (i.e., number of permanent pollinator individuals) or changes in their phenologies (i.e., length in days of the activity period of each pollinator species) as suggested by previous studies (CaraDonna et al. 2017; Resasco et al. 2021). Other factors, such as species traits or competitive interactions, may also influence these dynamics by shaping species' activity patterns or relative abundances, and could therefore indirectly contribute to these quantities, but are not considered here directly. The model of interaction rewiring (presented in Table 1 below) shows that rewiring among permanent species is mostly determined by changes in the abundance and phenology of permanent species, as well as by the amount of interaction changes due to species turnover. In particular, increased rewiring is heavily correlated with changes in the abundance of the permanent pollinators (see Figure 4A) and also positively correlated with changes in the phenology of permanent pollinators (Figure 4B), albeit less strongly. Finally, we also found that more interaction changes among the permanent species (i.e., rewiring) go hand in hand with more interaction changes involving transient species (Figure 4C), which suggests that the addition or loss of species does not merely alter the network composition but also indirectly reshuffles interaction patterns, reshaping who interacts with whom, as previously shown in experimental studies (Brosi and Briggs 2013). This underscores the cascading effects that species turnover can have on the network structure. For instance, when new species are added to a community or others are lost, the resulting changes may alter the probability of the remaining species interacting.

**TABLE 1:** Estimates and goodness‐of‐fit measures for the generalised model of interaction rewiring (quantified as GI, see methods). The independent variables included: Changes in pollinator abundances (Abn.), changes in pollinator phenologies (Pheno.), changes in plant abundances (Abn. P.), changes in plant phenologies (Pheno P.), and the number of interaction changes involving at least one transient species (C. trans., quantified as $GI_{T}$, see methods). For each model, we report the estimated coefficients and their standard error (in parentheses). Goodness‐of‐fit measures are also provided, including pseudo $R^{2}$ (ratio of the variance in predicted values to the variance in observed values).

Abn.	Pheno.	Abn. P	Pheno. P	C. trans.	*R* ^2^
0.47 (0.11)	0.14 (0.10)	−0.01 (0.06)	0.07 (0.07)	0.32 (0.08)	0.748

Despite being aware that the balance between gained and lost interactions may be influenced by imperfect detection during sampling (Weinstein and Graham 2017), such bias is likely limited to rare interactions. From the point of view of functional consequences, infrequent interactions are unlikely to contribute as much as frequent interactions to species persistence (Habel and Schmitt 2018; Redford 1992). More importantly, our results remain robust when excluding species observed only once per year (Figure S12), and we verified that sampling coverage was high and consistent across sites and years (Table S2).

## 4 Conclusion

The repeated calls to advance towards more predictive approximations in ecology point towards the necessity of incorporating the temporal nature of ecological interactions. Interaction turnover through time is hence pivotal to understanding and forecasting community responses to environmental changes (Godoy et al. 2024; Poisot et al. 2014; Song 2025). This shift emphasises moving beyond static network representations to consider how changes in species abundances, phenologies, and environmental conditions shape interaction patterns (Burkle and Alarcón 2011) and, concomitantly, community stability. In this work, we examine plant‐pollinator interactions over extended periods, highlighting the dynamic nature of these networks (Figure 2), Despite their variability, these communities retain a form of short‐term memory, with consecutive years being more similar to each other than those separated by longer intervals (Figure S2). Overall, our results indicate that the temporal dynamics of plant‐pollinator networks in our study closely resemble those observed in other reported systems (Chacoff et al. 2017; Miele et al. 2020; Olesen et al. 2011; Petanidou et al. 2008; Resasco et al. 2021). Therefore, we advocate that studying the consequences of the observed rewiring for the persistence of pollinators in our communities could be extrapolated to prior work. Our findings, in line with previous results (CaraDonna et al. 2017; Resasco et al. 2021), also reveal that shifts in pollinator species abundance and phenology are key drivers of interaction reorganisation (Figure 4), showcasing the adaptability of these ecological systems. In this context, our results show that generalist species at the core of mutualistic networks play a pivotal role in enhancing community persistence through their ability to rewire interactions year after year (Figure 3). This rewiring, though not optimal, acts as a buffer against environmental fluctuations and species loss, helping to maintain ecological stability. These potential networks are not realised in nature, however, likely due to ecological and evolutionary constraints resulting in forbidden links (Olesen et al. 2010). Those include phenological or spatial mismatches, or partner displacing due to competition relationships (Carvalheiro et al. 2014). Despite these constraints, we show that core interactions and their underlying drivers—phenology and abundance—emerge as essential mechanisms shaping community stability.

As global change continues to alter species abundances (Nicholson and Egan 2019; Potts et al. 2010; Vasiliev and Greenwood 2021) and phenologies (Bartomeus et al. 2011; CaraDonna et al. 2014; Freimuth et al. 2022), the ripple effects on species interactions and community persistence are likely to be profound (Tylianakis et al. 2008). Nonetheless, the inherent flexibility of real‐world networks provides substantial insurance, emphasizing interaction reorganization as a vital mechanism for the long‐term survival of mutualistic communities.

## Author Contributions

Virginia Domínguez‐Garcia, Oscar Godoy and Ignasi Bartomeus designed research. Francisco P. Molina and Ignasi Bartomeus did fieldwork. Francisco P. Molina identified species. Virginia Domínguez‐Garcia and Alfonso Allen‐Perkins developed code. Virginia Domínguez‐Garcia performed modelling work and analysed data. Virginia Domínguez‐Garcia and Ignasi Bartomeus performed research. Virginia Domínguez‐Garcia wrote the first draft and Oscar Godoy and Ignasi Bartomeus contributed substantially to revisions.

## Funding

This project has received funding from the European Union's Horizon 2021 research and innovation programme under the Marie Skłodowska‐Curie grant agreement No 101064340, and from the Emergia grant agreement DGP_EMEC_2023_00146 to Virginia Domínguez‐Garcia, TASTE Project (PID2021‐127607OB‐I00) to Oscar Godoy, Ignasi Bartomeus, Alfonso Allen‐Perkins, and Virginia Domínguez‐Garcia, and BeeFUN project (PCIG 14‐GA‐2013‐631653) to Ignasi Bartomeus.

## Supporting information


**Data S1:** ele70293‐sup‐0001‐Supinfo.pdf.

## Data Availability

The empirical data and codes for the study are uploaded in Zenodo and can be accessed here.
