# Supplementary material for: Plant‐Pollinator Interaction Rewiring Boosts Year‐to‐Year Community Persistence
Source: Ecol Lett. 2026 Jan 12;29(1):e70293. doi: 10.1111/ele.70293 (PMC12794140; doi:10.1111/ele.70293)
Supplement: Supplementary file 1 — Data S1: ele70293‐sup‐0001‐Supinfo.pdf. [file ELE-29-0-s001.pdf]

Supporting Information for

Plant-pollinator interaction rewiring boosts year to year community persistence

Virginia Domínguez-Garcia, Francisco P. Molina, Alfonso Allen-Perkins, Oscar Godoy, and Ignasi Bartomeus

Virginia Domínguez-García
E-mail: domgarvir@gmail.com

This PDF file includes:

Figs. S1 to S13
Tables S1 to S5
SI References

1. Supplementary methods

A. Database and sampling coverage. .

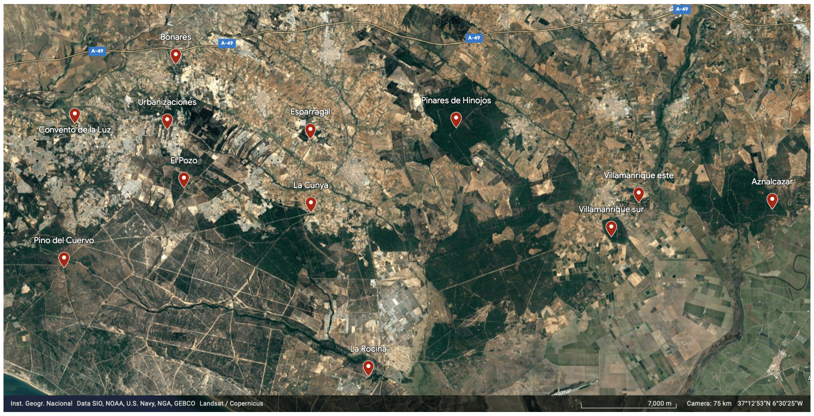

Fig. S1. Map of the 12 sites studied along a fragmentation gradient. Reproduced from Google Earth Pro 7.3.6.9345.(December 29, 2022). Doñana Region, SW Spain. 37° 12' 53"N, 6° 30' 25"W, Eye alt 7000 m. Borders and labels; places layers. Inst. Geogr. Nacional, SIO, NOAA, U.S. Navy, NGA, GEBCO. Landsat/Copernicus. http://www.google.com/earth/index.html (Accessed August 2, 2023)

Site	Plant sp.	Pollinator sp.	Interactions
Aznalcazar	29	119	275
La rocina	30	114	218
Pino del cuervo	34	112	231
Convento de la luz	27	111	244
Urbanizaciones	26	106	234
Villamanrique este	25	104	223
Bonares	23	99	168
El pozo	27	94	182
Villamanrique sur	21	78	140
La cuña	28	73	149
Pinares de hinojos	32	62	119
Esparragal	23	54	92

Table S1. Summary statistics of the interaction networks in our study. From left to right columns indicate site name, number of different plant species recorded, number of pollinator species recorded, and number of interactions recorded (not frequency) trough all the years.

	2015	2016	2017	2018	2019	2020	2021	2022	mean	std
Assemblage										
aznalcazar	0.77	0.86	0.8	0.8	0.78	0.83	0.89	0.78	0.81	0.04
bonares	0.53	0.7	0.78	0.76	0.6	0.74	0.85	0.62	0.7	0.11
convento de la luz	0.82	0.74	0.76	0.77	0.85	0.74	0.9	0.75	0.79	0.06
el pozo	0.73	0.74	0.65	0.61	0.55	0.71	0.78	0.66	0.68	0.08
esparragal	0.58	0.64	0.78	0.75	0.78	0.69	0.82	0.36	0.68	0.15
la cuña	0.64	0.6	0.71	0.56	0.83	0.86	0.78	0.7	0.71	0.11
la rocina	0.62	0.73	0.93	0.73	0.78	0.86	0.92	0.72	0.79	0.11
pinares de hinojos	0.61	0.76	0.87	0.76	0.83	0.72	0.83	0.6	0.75	0.1
pino del cuervo	0.62	0.57	0.84	0.84	0.8	0.82	0.84	0.77	0.76	0.11
urbanizaciones	0.64	0.77	0.87	0.85	0.8	0.83	0.92	0.81	0.81	0.09
villamanrique este	0.54	0.7	0.71	0.25	0.82	0.72	0.9	0.82	0.68	0.21
villamanrique sur	0.58	0.67	0.73	0.57	0.76	0.66	0.73	0.68	0.67	0.07
mean	0.64	0.71	0.79	0.69	0.76	0.77	0.85	0.69	-	-
std	0.09	0.08	0.08	0.17	0.09	0.07	0.06	0.13	-	-

Table S2. Sample coverage of the different sites of the study for each year. The "mean" column contains the average value of the sample coverage for each site over time, while the "mean" row contains the average sample coverage for a given year across all sites.

B. Quantification of interaction and species turnover. .

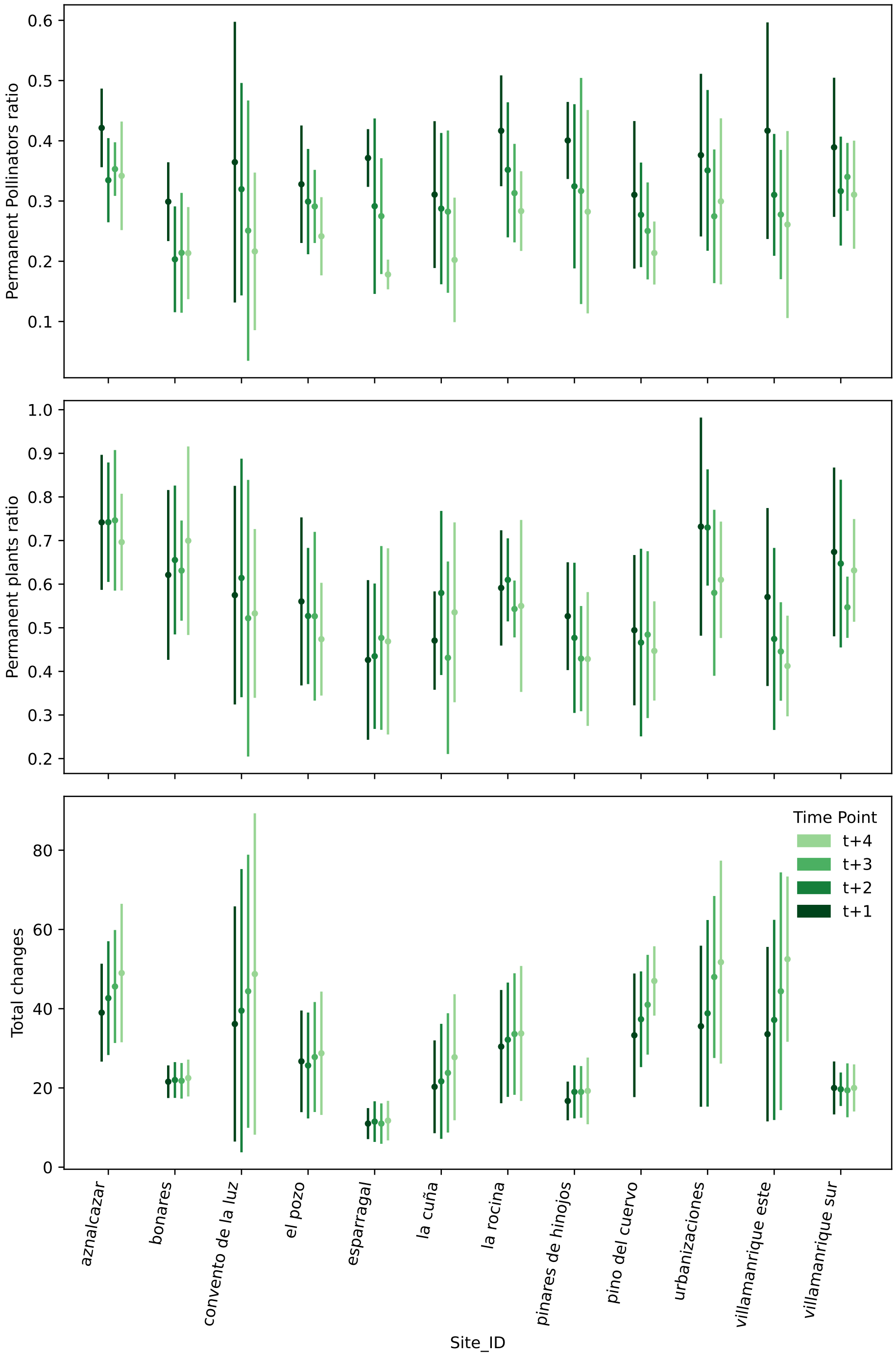

Fig. S2. Temporal comparison of network properties across increasing time lags. Each point represents the average value of a given metric across sites for a specific time window, and error bars indicate the standard deviation. Note that the number of available comparisons decreases as the time lag increases: for instance, the 8 networks at a given site can be compared 7 times if we consider t vs. $t+1$, 6 times for t vs. $t+2$, and so on, down to only 4 comparisons for t vs. $t+4$. A) Change in the ratio of permanent to transient pollinators as the temporal gap between networks increases. B) Change in the ratio of permanent to transient plants with increasing temporal distance between networks. C) Change in total interaction turnover as networks further apart in time are compared.

C. Quantification of rewiring and species turnover contribution to interaction turnover. Interaction turnover is decomposed additively into two components following the framework presented in (1, 2): differences due to species composition turnover (β_{ST}) and differences in the interactions among shared species, or rewiring (β_{OS}), such that

$$\beta_{WN} = \beta_{ST} + \beta_{OS}$$

While this framework is useful and has been widely adopted, it infers the contribution of species turnover indirectly—by subtracting the dissimilarity among shared species (rewiring) from the total interaction dissimilarity—rather than quantifying it explicitly. In contrast, our approach directly classifies each interaction change as either rewiring (between permanent species) or species turnover (involving at least one transient species).

To compare both methods, we applied them to one of the networks in our study (Aznalcázar 2015–2016; see Fig. S3). We first quantified the number of permanent interactions (i.e. exist in t and $t+1$), named a in (1), and represented by green squares in Fig. S3. We also quantified how many links exist only in t, (represented by blue squares, and named b). A distinction can be made according to the nature of the species involved; they can be lost links among permanent species (represented by hatched squares, and quantified as b'), or can involve a transient species that disappears (plain squares, and quantified as b"). We also quantified how many new links appear in $t + 1$ (yellow squares, quantified as c), and then see how many of those are new links between permanent species (dashed squares, quantified as c'), and how many new links involve transient species (full squares, quantified as c").

Following the Poisot et al. (2012) framework of beta-partitioning, 52% of interaction turnover was attributed to β_{OS} (i.e.,rewiring). However, our direct approach revealed that, in contrast, only 24% of new interactions occurred among permanent species—i.e., rewiring (See Fig. S3). Extending this comparison to all networks in our study (Fig. S4A), we found that the standard β_{OS} approach consistently overestimates rewiring, often assigning 60% of interaction turnover to it. In contrast, our direct quantification shows that rewiring typically accounts for only 20% of interaction turnover, with the majority of interaction changes being caused by species turnover. Despite this discrepancy, both metrics are strongly correlated across networks (Fig. S4B), suggesting they are similarly effective for comparative purposes among networks. Still, when the goal is to quantify the amount of interaction turnover due to rewiring or species turnover in a particular network, we believe our method offers a more accurate picture. In fact, the original author of the method already warns that some of the components mix turnover and rewiring as stated in a recent paper that says "β_{ST} captures both turnover and rewiring mechanisms, and therefore β_{ST}/β_{WN} should be interpreted more thoroughly"(3). In addition, our result aligns with previous results highlighting the dominant role of species turnover in driving temporal changes in mutualistic networks, particularly at inter-annual scales.(4).

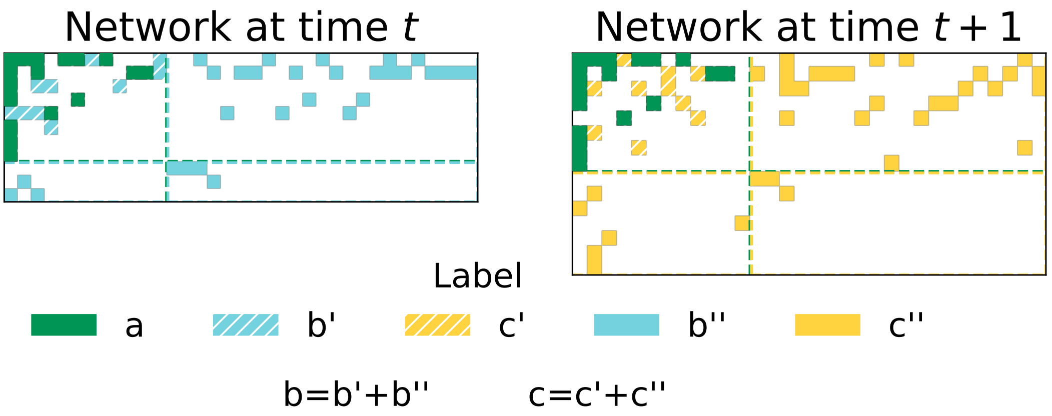

	Name	Value
Interactions in both networks	a	17
Interactions only in Network 2	b	44
Interactions only in Network 1	c	39
Interactions among permanent species only in Network 2	b’	10
Interactions among permanent species only in Network 1	c’	10
Interactions only in Network 2 that involve one transient species	b’’	34
Interactions only in Network 1 that involve one transient species	c’’	29

	Formula	Value	Percent
β_{WN}	$2\,(a+b+c)/(2\,a+b+c)\ -1$	0.71	1.0
β_{OS}	$2\,(a+b'+c')/(2\,a+b'+c')\ -1$	0.37	0.52
β_{ST}	$\beta_{WN}-\beta_{OS}$	0.34	0.48
Total interaction turnover	$b+c=b'+b''+c'+c''$	83.0	1.0
Interaction turnover caused by rewiring	$b'+c'$	20.0	0.24
Interaction turnover caused by sp. turnover	$b''+c''$	63.0	0.76

Fig. S3. S8.pngction turnover quantification using two annual networks in one of the study sites (Aznalcázar in 2015 and 2016) as an example.

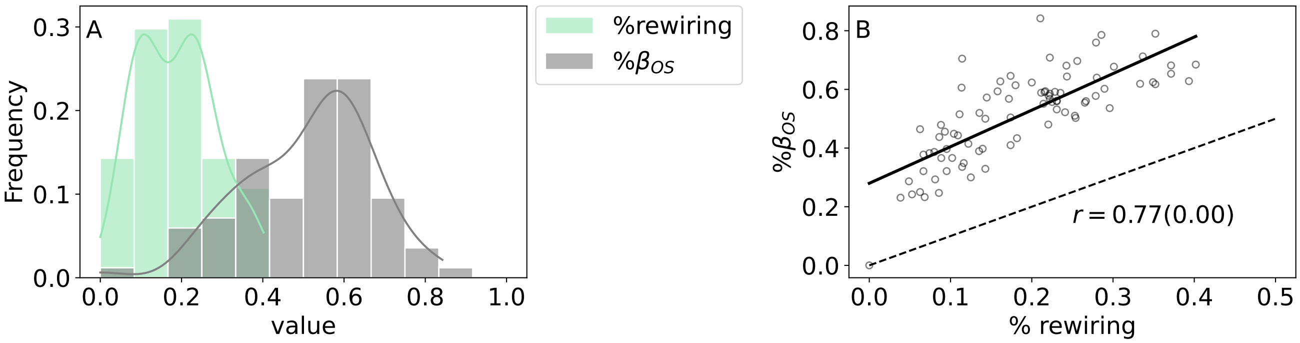

Fig. S4. Comparison of the contribution of rewiring considering different metrics. A) Distribution of values of interaction turnover due to rewiring using two different metrics: direct quantification (in light green, as we follow in the main text) and using the methodology presented in (1, 2). B) Comparison of the % of interaction turnover caused by rewiring quantified using β_{OS} or directly by counting the changes.

D. Deriving effective biotic interaction matrices. In order to disentangle plant and pollinator systems we apply the transformation presented in (5), multiplying both left sides of equation 1 by $T = 1 + \Gamma C^{-1}$. The transformations allow going from this system where plants and pollinators are entangled ($N_P + N_A$ equations)

$$\begin{bmatrix} r^P \\ r^A \end{bmatrix} = \Bigg(\overbrace{\underbrace{\begin{bmatrix} \alpha^P & 0 \\ 0 & \alpha^A \end{bmatrix}}_{\mathbf{C}} - \underbrace{\begin{bmatrix} 0 & \gamma^P \\ \gamma^A & 0 \end{bmatrix}}_{\mathbf{\Gamma}}}^{\mathbf{A}} \Bigg) \begin{bmatrix} P \\ A \end{bmatrix} = -\hat{A} \begin{bmatrix} P \\ A \end{bmatrix} \qquad [1]$$

to these two disentangled systems, one for plants (with N_P equations) and another for pollinators (with N_A equations)

$$\begin{bmatrix} r^P + \gamma^P(\alpha^A)^{-1} r^A \\ r^A + \gamma^A(\alpha^P)^{-1} r^P \end{bmatrix} = \begin{bmatrix} \alpha^P - \gamma^P(\alpha^A)^{-1}\gamma^A & 0 \\ 0 & \alpha^A - \gamma^A(\alpha^P)^{-1}\gamma^P \end{bmatrix} \begin{bmatrix} P \\ A \end{bmatrix} \qquad [2]$$

that represents the effective intra-guild interaction once the competition and mutualistic effects have been taken into account

$$\begin{bmatrix} r'_P \\ r'_A \end{bmatrix} = \begin{bmatrix} \alpha'_P & 0 \\ 0 & \alpha'_A \end{bmatrix} \begin{bmatrix} P \\ A \end{bmatrix} \qquad [3]$$

In this new framework r'_P and r'_A are called effective intrinsic growth rates and α'_A and α'_P effective interaction. These two matrices are the ones we use to quantify the structural stability of the pollinator community and of the plant community.

E. LMM residuals. .

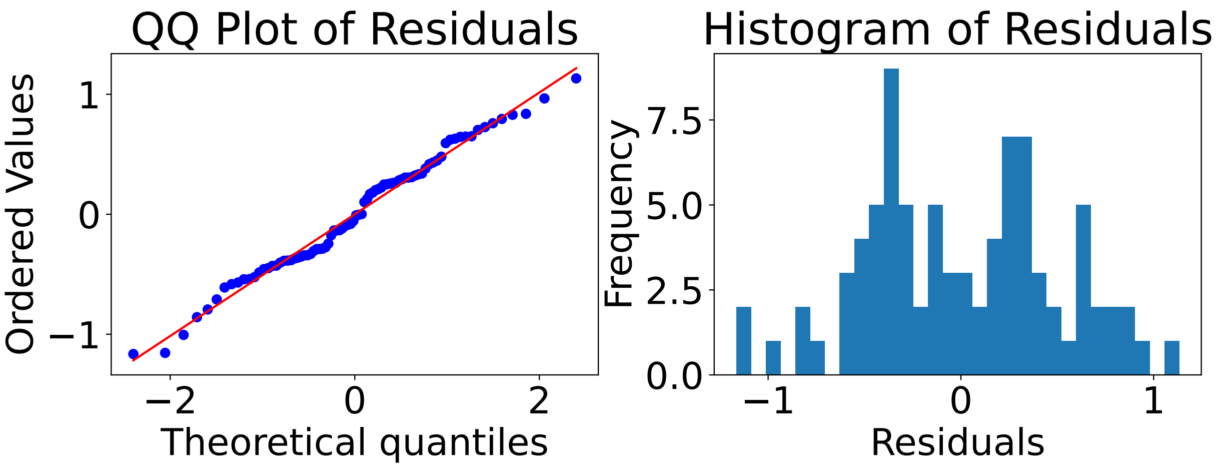

Fig. S5. residuals of the LMM. Left panel: Q–Q plot showing that the residuals closely follow the theoretical normal distribution, indicating that the assumption of normality is met. Right panel: Histogram of residuals demonstrating a roughly symmetric and unimodal distribution, supporting the validity of the linear model assumptions.

2. Supplementary results

A. Landscape effect on interaction turnover and rewiring. .

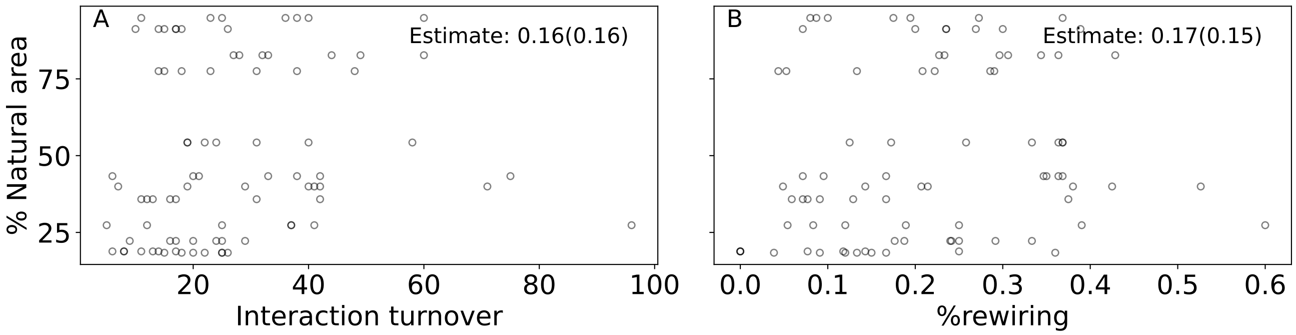

Fig. S6. Landscape intensification vs interaction turnover and amount of rewiring. A) Scatter plot of the amount of natural area (measured as the percentage of area inside a buffer of 1km around the site composed by natural environment, i.e pine forest or shrubs) vs the total amount of interaction turnover in the site. B) Scatter plot of the amount of natural area vs the percentage of interaction turnover caused by rewiring. Each point represents one annual network. The text includes the estimate and estandard error (in parentheses) of a linear mixed model using site as a random factor, to account for the nested nature of the data.

B. Randomized and potential networks. .

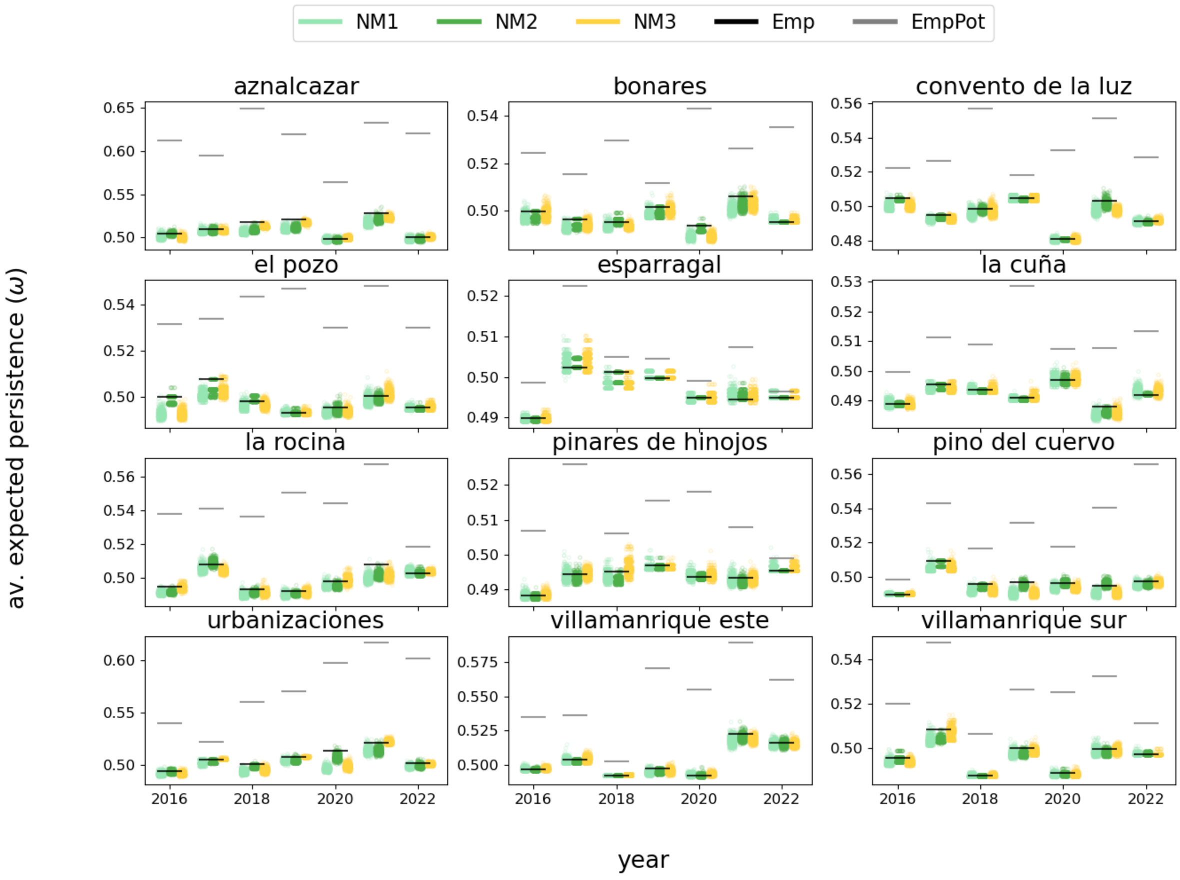

Fig. S7. Structural stability (ω) of the pollinators in different sites and years. Horizontal black lines mark the value of ω of the empirical networks, horizontal grey lines the value of ω of the potential network of interactions for the species present in a given year, and dots represent the values of 500 randomized networks using the random null model (light green), random rewiring null model (green), and the random turnover null model (yellow). We see that while the empirical network is usually better than the randomizations, it is not optimal (there are some random configurations that have higher values of ω). Both the empirical and the best randomizations are in any case far from the value of ω of the potential network of interactions.

C. Optimized networks and their structure. We include below the example of two "optimized" networks generated using the least restrictive of our null models (random model) for one of our study sites, Aznalcázar (Fig. S8). We use quotation marks deliberately to emphasize that we are not applying a formal optimization algorithm. Instead, we generate a large ensemble of randomized networks and select those with the highest structural stability to examine the features they have in common. As shown in the figure, it is difficult—by eye alone—to identify which structural properties distinguish these high-performing networks. To better understand the drivers of increased structural stability, we quantified several network-level metrics that are commonly associated with stability in mutualistic systems: nestedness (6), degree–degree correlations (assortativity) (7), pollinator degree heterogeneity(8), and the mean number of shared mutualistic partners.

We quantified nestedness (ν) following (9), and degree assortativity (r) following (7). Degree heterogeneity (DH_A) was calculated as the ratio of the standard deviation to the mean number of mutualistic partners among pollinators (σ_A/μ_A), with values above 1 indicating high disparity in partner number. The number of shared mutualistic partners (SP) was defined as the average number of plant species shared between all pairs of pollinators).

Importantly, we compared these properties under controlled conditions—keeping constant the network size, the number of permanent and transient interactions, and ensuring that all species had at least one interaction. Our results (Fig. S9) reveal that nestedness and disassortativity (i.e., negative degree–degree correlations) both contribute positively to structural stability. However, the strongest signal comes from degree heterogeneity (Fig. S9C), which consistently correlates with higher stability. These more heterogeneous networks inherently lead to more shared mutualistic partners (Fig. S9D), a pattern that emerges naturally when all other structural features are held constant. These shared interaction pathways appear to be a key determinant of enhanced structural stability in our communities. Finally, it is worth noting that some of the interactions present in these "optimized" networks could not occur in nature due to mismatched phenologies—i.e., the interacting species were not temporally co-occurring. To distinguish temporally feasible from infeasible links, we highlight possible interactions—those between species with overlapping phenologies—in light purple in figure R.S8.

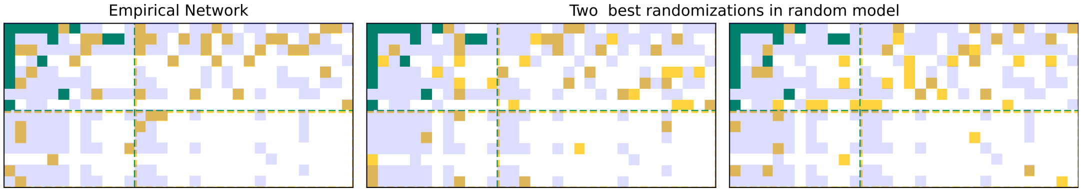

Fig. S8. Optimized networks. Left panel: Empirical network of Aznalcázar in 2016. Permanent interactions (i.e., present in both 2015 and 2016) are shown in green, while transient interactions (i.e., unique to 2016) are shown in yellow. Purple-shaded cells indicate all possible interactions between species that overlapped phenologically during 2016. Center and right panels: The two highest-persistence networks among 100 randomized networks generated using the random null model. Note that some interactions in these optimized networks involve species without overlapping phenologies in the field and would therefore not have occurred in the field.

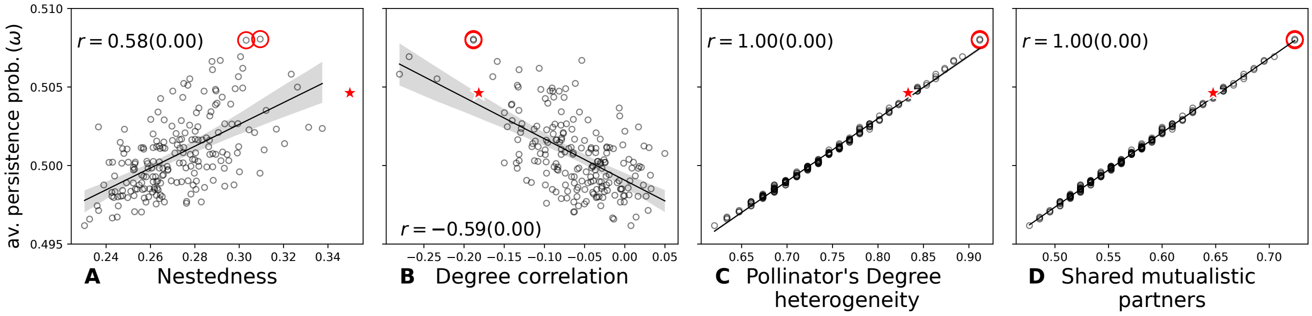

Fig. S9. Structural features fostering pollinator persistence. Scatter plots showing the relationship between pollinators' average probability of persistence (quantified as ω) and four structural metrics of the mutualistic network: (a) nestedness, (b) degree assortativity, (c) degree heterogeneity, and (d) number of shared mutualistic partners. Each point represents one of the 100 randomized networks generated using the random null model. The empirical network is marked with a red star, and the two highest-persistence randomized networks are highlighted with red circles. Shaded areas represent the 95% confidence intervals of the regression estimates (black lines). Pearson correlation coefficients and their corresponding p-values are shown in each pane

Site ID	Year	Metric<br>Null model	ν	σ_A	SP	Value<br>r
aznalcazar	2016	1	0.567	0.998	0.999	-0.689
		2	0.609	0.997	0.998	-0.616
		3	0.534	0.997	0.999	-0.589
	2017	1	0.433	0.998	0.999	-0.535
		2	0.448	0.998	0.999	-0.673
		3	0.552	0.999	1.000	-0.727
	2018	1	0.516	0.997	0.999	-0.412
		2	0.318	0.997	0.998	-0.566
		3	0.338	0.997	0.997	-0.227
	2019	1	0.346	0.997	0.998	-0.288
		2	0.394	0.997	0.999	-0.708
		3	0.401	0.996	0.997	-0.449
	2020	1	0.528	0.998	0.999	-0.089
		2	0.479	0.998	0.999	-0.458
		3	0.568	0.999	0.999	-0.475
	2021	1	0.387	0.996	0.997	-0.158
		2	0.402	0.993	0.994	-0.576
		3	0.354	0.994	0.995	-0.191
	2022	1	0.215	0.996	0.998	-0.180
		2	0.429	0.992	0.994	-0.471
		3	0.461	0.997	0.998	-0.329
bonares	2016	1	0.558	0.995	1.000	-0.462
		2	0.512	0.997	1.000	-0.631
		3	0.598	0.997	1.000	-0.458
	2017	1	0.517	0.995	1.000	-0.313
		2	0.648	0.997	1.000	-0.759
		3	0.454	0.996	1.000	-0.432
	2018	1	0.448	0.993	0.999	-0.085
		2	0.774	0.998	1.000	-0.391
		3	0.433	0.995	0.999	-0.394
	2019	1	0.587	0.996	0.999	-0.593
		2	0.310	0.996	0.999	-0.832
		3	0.464	0.995	1.000	-0.619
	2020	1	0.533	0.996	1.000	-0.379
		2	0.876	0.999	1.000	-0.594
		3	0.539	0.995	1.000	-0.259
	2021	1	0.503	0.997	1.000	-0.536
		2	0.600	0.998	1.000	-0.506
		3	0.638	0.997	1.000	-0.420
	2022	1	0.490	0.995	0.998	0.018
		2	0.702	0.437	–	0.526
		3	0.402	0.987	0.999	0.044
convento de la luz	2016	1	0.602	0.997	1.000	-0.542
		2	0.494	0.999	1.000	0.068
		3	0.542	0.998	1.000	-0.476
	2017	1	0.634	0.996	1.000	-0.474
		2	0.867	1.000	1.000	-0.864
		3	0.483	0.998	1.000	-0.285
	2018	1	0.643	0.997	0.999	-0.405
		2	0.759	0.999	1.000	-0.329
		3	0.780	0.997	1.000	-0.153
	2019	1	0.663	0.998	1.000	-0.691
		2	0.403	1.000	1.000	-0.197
		3	1.000	0.998	1.000	-0.773
	2020	1	0.558	0.996	1.000	-0.286
		2	0.524	1.000	1.000	0.330

Continued on next page

Site ID	Year	Metric<br>Null Model	ν	σ_A	SP	Value<br>r
		3	0.509	0.996	1.000	-0.264
	2021	1	0.491	0.995	0.997	-0.087
		2	0.362	0.995	0.995	-0.536
		3	0.623	0.996	0.997	-0.474
	2022	1	0.252	0.995	0.998	-0.123
		2	0.347	0.993	0.993	-0.062
		3	0.321	0.996	0.997	-0.273
el pozo	2016	1	0.474	0.996	0.999	-0.447
		2	0.378	0.999	0.999	-0.960
		3	0.517	0.996	0.999	-0.567
	2017	1	0.627	0.994	1.000	-0.610
		2	0.306	0.995	1.000	-0.804
		3	0.504	0.997	1.000	-0.549
	2018	1	0.488	0.995	0.999	-0.230
		2	0.357	0.999	0.999	-0.676
		3	0.526	0.993	1.000	-0.219
	2019	1	0.433	0.985	0.998	-0.105
		2	0.165	0.991	0.999	-0.568
		3	0.409	0.991	0.999	-0.346
	2020	1	0.466	0.997	0.999	-0.282
		2	0.277	0.995	0.998	-0.663
		3	0.509	0.996	0.999	-0.498
	2021	1	0.527	0.995	0.999	-0.312
		2	0.311	0.992	0.994	-0.510
		3	0.380	0.998	0.999	-0.419
	2022	1	0.318	0.995	0.998	-0.199
		2	0.078	0.994	0.998	-0.202
		3	0.602	0.991	0.999	-0.125
esparragal	2016	1	0.573	0.992	1.000	-0.283
		2	0.551	1.000	1.000	-0.355
		3	0.513	0.993	1.000	-0.161
	2017	1	0.479	0.997	1.000	-0.539
		2	0.811	0.999	0.999	-0.914
		3	0.582	0.995	1.000	-0.626
	2018	1	0.711	0.988	1.000	-0.730
		2	0.473	1.000	1.000	-0.863
		3	0.529	0.990	1.000	-0.756
	2019	1	0.341	0.999	0.999	-0.317
		2	–	–	–	–
		3	0.375	0.998	1.000	-0.277
	2020	1	0.788	0.995	1.000	-0.411
		2	–	–	–	–
		3	0.657	0.987	0.999	-0.571
	2021	1	0.340	0.996	0.999	-0.477
		2	0.423	0.996	0.999	-0.895
		3	0.477	0.996	0.999	-0.383
	2022	1	0.519	0.999	0.999	-0.421
		2	–	–	–	–
		3	0.595	1.000	1.000	-0.438
la cuña	2016	1	0.539	0.995	1.000	-0.049
		2	0.558	0.999	1.000	-0.002
		3	0.504	0.993	1.000	-0.170
	2017	1	0.447	0.994	1.000	-0.402
		2	0.891	1.000	1.000	-0.358
		3	0.590	0.991	1.000	-0.298
	2018	1	0.523	0.995	1.000	-0.089

Continued on next page

Site ID	Year	Metric<br>Null Model	ν	σ_A	SP	Value<br>r
		2	0.628	1.000	1.000	-0.163
		3	0.553	0.984	1.000	-0.223
	2019	1	0.444	0.996	1.000	-0.067
		2	0.404	1.000	1.000	0.132
		3	0.663	0.988	1.000	-0.228
	2020	1	0.531	0.990	1.000	-0.653
		2	0.463	0.994	0.999	-0.623
		3	0.616	0.995	1.000	-0.758
	2021	1	0.536	0.996	0.999	-0.338
		2	0.267	0.997	0.998	-0.374
		3	0.510	0.996	0.999	-0.255
	2022	1	0.483	0.995	0.999	-0.233
		2	0.210	0.988	0.988	0.093
		3	0.344	0.996	0.999	-0.082
la rocina	2016	1	0.306	0.997	0.999	-0.319
		2	0.224	0.995	0.998	-0.670
		3	0.430	0.998	1.000	-0.654
	2017	1	0.480	0.997	0.999	-0.305
		2	0.436	0.998	0.999	-0.518
		3	0.536	0.997	0.999	-0.621
	2018	1	0.532	0.997	0.999	-0.230
		2	0.221	0.998	0.999	-0.543
		3	0.463	0.996	0.999	-0.177
	2019	1	0.475	0.994	0.999	-0.479
		2	0.191	0.996	0.998	-0.270
		3	0.568	0.994	0.999	-0.617
	2020	1	0.384	0.996	0.999	-0.577
		2	0.475	0.996	0.999	-0.706
		3	0.531	0.996	1.000	-0.815
	2021	1	0.451	0.995	0.997	-0.301
		2	0.453	0.997	0.998	-0.600
		3	0.347	0.997	0.999	-0.297
	2022	1	0.439	0.995	0.997	-0.262
		2	0.166	0.995	0.999	-0.421
		3	0.462	0.994	0.995	-0.238
pinares de hinojos	2016	1	0.574	0.995	1.000	-0.101
		2	0.577	0.995	1.000	0.371
		3	0.684	0.991	1.000	0.043
	2017	1	0.603	0.996	1.000	-0.214
		2	0.497	0.996	1.000	-0.032
		3	0.660	0.998	1.000	-0.505
	2018	1	0.605	0.994	1.000	-0.538
		2	0.452	0.996	1.000	-0.269
		3	0.482	0.997	1.000	-0.397
	2019	1	0.399	0.986	0.999	-0.252
		2	0.271	0.999	0.999	-0.083
		3	0.393	0.994	0.998	-0.756
	2020	1	0.511	0.991	0.999	-0.431
		2	0.715	0.998	0.999	-0.722
		3	0.473	0.994	1.000	-0.463
	2021	1	0.609	0.995	0.999	-0.375
		2	0.444	0.996	0.999	-0.469
		3	0.366	0.995	0.999	-0.346
	2022	1	0.482	0.995	0.998	-0.236
		2	0.868	–	–	0.872
		3	0.335	0.996	0.999	-0.194

Continued on next page

Site ID	Year	Metric<br>Null Model	ν	σ_A	SP	Value<br>r
pino del cuervo	2016	1	0.474	1.000	1.000	-0.060
		2	0.969	–	–	-0.113
		3	0.543	0.998	1.000	-0.270
	2017	1	0.373	0.998	0.999	-0.302
		2	0.894	1.000	1.000	-0.996
		3	0.537	0.997	1.000	-0.520
	2018	1	0.556	0.997	1.000	-0.547
		2	0.600	1.000	1.000	-0.620
		3	0.478	0.998	1.000	-0.549
	2019	1	0.541	0.996	0.998	-0.499
		2	0.319	0.998	0.999	-0.785
		3	0.355	0.996	0.998	-0.510
	2020	1	0.218	0.996	0.998	-0.434
		2	0.088	0.997	0.999	-0.705
		3	0.436	0.993	0.998	-0.486
	2021	1	0.471	0.996	0.998	-0.268
		2	0.243	0.998	0.999	-0.620
		3	0.458	0.996	0.998	-0.191
	2022	1	0.387	0.994	0.998	-0.257
		2	0.215	0.996	0.998	-0.172
		3	0.217	0.996	0.998	-0.419
urbanizaciones	2016	1	0.440	0.997	1.000	-0.404
		2	0.419	0.998	1.000	-0.326
		3	0.478	0.997	1.000	-0.480
	2017	1	0.598	0.994	1.000	-0.250
		2	0.439	1.000	1.000	-0.409
		3	0.287	0.998	0.998	0.163
	2018	1	0.626	0.996	0.999	-0.402
		2	0.757	0.999	1.000	-0.878
		3	0.715	0.997	1.000	-0.505
	2019	1	0.328	0.997	0.999	-0.384
		2	0.314	0.998	0.999	-0.330
		3	0.472	0.991	0.991	-0.063
	2020	1	0.618	0.997	0.999	-0.447
		2	0.586	0.998	0.999	-0.440
		3	0.706	0.998	1.000	-0.298
	2021	1	0.540	0.996	0.998	-0.178
		2	0.422	0.995	0.996	-0.385
		3	0.532	0.992	0.992	-0.392
	2022	1	0.256	0.996	0.997	-0.200
		2	0.031	0.997	0.998	-0.299
		3	0.315	0.997	0.997	-0.378
villamanrique este	2016	1	0.335	0.997	0.999	-0.175
		2	-0.010	0.998	0.998	-0.132
		3	0.468	0.997	0.999	-0.412
	2017	1	0.422	0.997	0.999	-0.310
		2	0.130	0.999	0.999	-0.354
		3	0.532	0.995	0.999	-0.208
	2018	1	0.887	–	–	–
		2	–	–	–	–
		3	0.676	1.000	1.000	-0.129
	2019	1	0.317	0.996	0.999	-0.037
		2	0.650	0.996	0.998	-0.273
		3	0.591	0.996	0.999	-0.158
	2020	1	0.401	0.995	0.999	-0.159
		2	0.079	0.990	0.994	-0.293

Continued on next page

Site ID	Year	Metric<br>Null Model	ν	σ_A	SP	Value<br>r
		3	0.408	0.995	0.998	-0.267
	2021	1	0.596	0.995	0.997	-0.261
		2	0.350	0.989	0.990	-0.231
		3	0.557	0.993	0.993	-0.310
	2022	1	0.653	0.997	0.999	-0.565
		2	0.668	0.997	0.998	-0.469
		3	0.581	0.998	0.998	-0.587
villamanrique sur	2016	1	0.519	0.996	0.999	-0.520
		2	0.577	0.996	1.000	-0.782
		3	0.430	0.994	0.999	-0.445
	2017	1	0.426	0.994	0.999	-0.228
		2	-0.004	0.995	0.998	-0.546
		3	0.554	0.995	1.000	-0.560
	2018	1	0.339	0.995	0.999	-0.275
		2	0.036	0.998	0.998	-0.206
		3	0.382	0.993	0.999	-0.312
	2019	1	0.410	0.994	0.999	-0.319
		2	0.371	0.995	0.999	-0.600
		3	0.503	0.996	0.998	-0.495
	2020	1	0.546	0.981	0.999	-0.111
		2	0.522	0.993	1.000	-0.256
		3	0.464	0.990	0.999	-0.284
	2021	1	0.572	0.994	0.999	-0.367
		2	0.462	0.996	0.999	-0.545
		3	0.638	0.997	0.999	-0.399
	2022	1	0.549	0.994	0.999	-0.471
		2	0.246	0.998	0.998	-0.301
		3	0.291	0.994	0.995	-0.107

Table S3. Correlation between av. pollinaotr persistnce probability (ω) with four structural features of the ecological networks: Nestedness (ν), Pollinator degree heterogeneity (σ_A), number of shared mutualistic partners (SP), and degree assortativity (r)

3. Robustness of the results

A. Robustness of results to singleton species removal. .

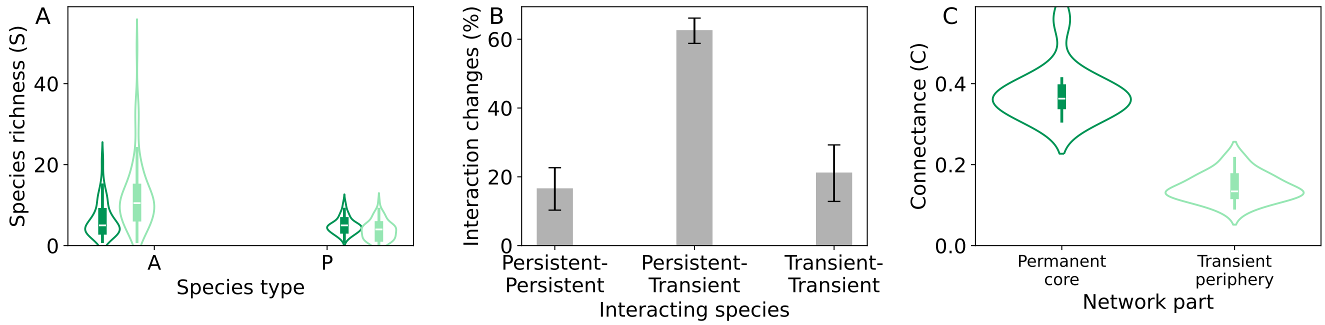

Fig. S10. A) Distribution of permanent species richness (i.e. number of species that remain for two consecutive years, in deep green) and of transient species richness (in light green) for pollinators and plants in our studied annual networks without considering singleton species. B) Distribution of ecological interaction changes according to the permanent/transient nature of the interaction partners. The height of the bar indicates the average value across the annual networks without singleton species, and the error bars represent one standard deviation. E) Density of interactions (i.e. connectance) among permanent species compared to its value in the rest of the network, not considering the singleton species.

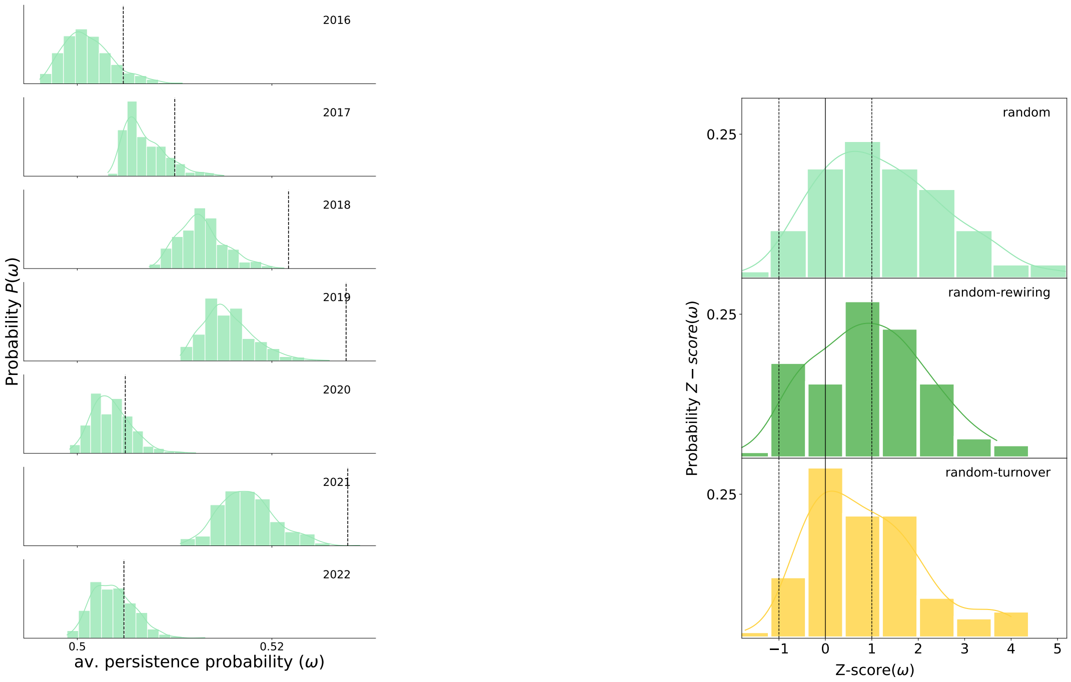

Fig. S11. Comparative of the expected pollinator persistence in empirical networks without considering singleton species compared with their randomised counterparts. Left) Distribution of the values of expected pollinator persistence (quantified as ω) for 500 randomizations using the "random null model", for each year in one of our study sites (Aznalcazar). The black dashed line indicates the value of the empirical network in each year. Rigth) Distributions of Z-score for the average persistence of pollinators (ω) across the 84 empirical networks under the three null models, each shown in a different color. The solid vertical lines indicate $Z = 0$, while the dashed lines mark $Z = \pm 1$.

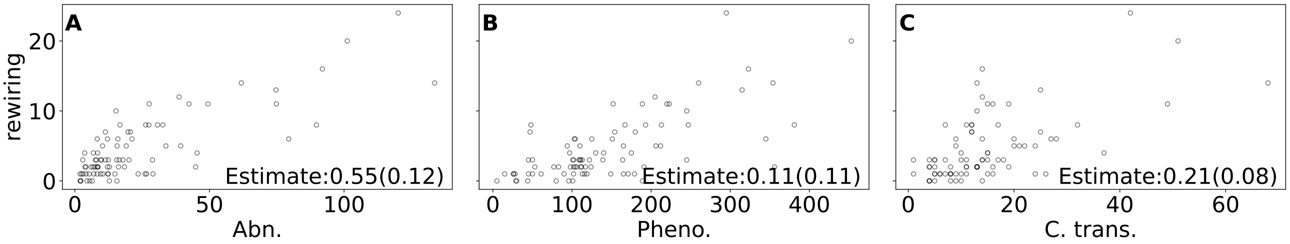

Fig. S12. Drivers of interaction rewiring in networks without singleton species. Rewiring vs the three explanatory variables retained in the model of rewiring: A) changes in abundance of permanent pollinator species, B) amount of phenological changes in the permanent pollinator species, and C) number of interaction changes caused by species turnover. Each point represents one of the 84 networks (without considering singleton species) in the study. Values in each panel indicate the model estimate and its standard error (in parentheses), obtained from the linear mixed-effects model shown in Table R.1 below.

Abn.	Pheno.	Abn. P	Pheno. P	C. trans.	R2	AIC	BIC
0.55(0.12)	0.11(0.11)	0.03(0.07)	0.05(0.08)	0.21(0.08)	0.701	174.666	194.112

Table S4. Estimates and goodness-of-fit measures for the model of interaction rewiring exclusing singleton species. The independent variables included: changes in pollinator abundances (Abn.), changes in pollinator phenologies (Pheno.), changes in plant abundances (Abn. P.), changes in plant phenologies (Pheno P.), and the number of interaction changes involving at least one transient species (C. trans.). For each model, we report the estimated coefficients and their standard error (in parentheses). Goodness-of-fit measures are also provided, including pseudo R^2 (ratio of the variance in predicted values to the variance in observed values), Akaike Information Criterion (AIC), and Bayesian Information Criterion (BIC).

76 ## B. Symmetric vs asymmetric quantification of interaction and species turnover. .

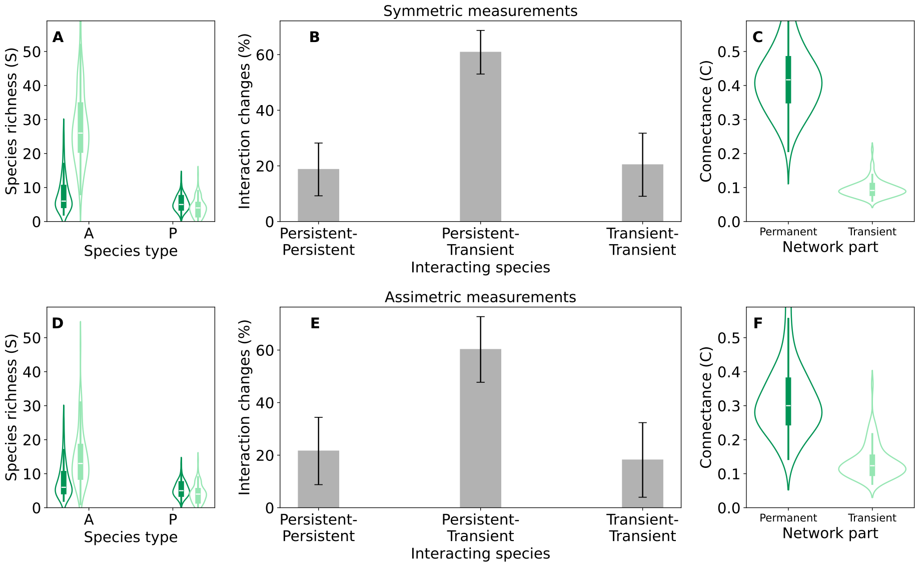

Fig. S13. Comparative of metrics of species and interaction turnover considering the symmetric approach (considering the full networks in t and $t+1$, upper row) and the asymmetric approach we take in the manuscript (focusing on network at time $t+1$). A and D: Permanent and transient species richness in the annual networks in the study. B and E: Distribution of ecological interaction changes according to the permanent/transient nature of the interaction partners. C and F: Distribution of the density of interactions (i.e. connectance) in the permanent species core (formed by the permanent species) compared to its value in the transient periphery (formed by transient species).

77 ## C. Robustness of results to dynamical model parameter choice. .

Table S5. Percentage of networks with a Z-score above one in the three null models implemented in our study as a function of the dynamical parameters of the mutualistic model: intra-guild mean-field competition (α), average mutualistic gain(γ_0), and mutualistic trade-off (δ).

α	γ_0	δ	NM1	NM2	NM3
0.001	0.100	0.000	59.52	58.33	44.05
		0.200	57.14	59.52	44.05
		0.500	50.00	57.14	39.29
		0.700	42.86	57.14	30.95
	0.110	0.000	59.52	58.33	44.05
		0.200	57.14	61.90	44.05
		0.500	50.00	57.14	39.29
		0.700	42.86	54.76	30.95
	0.120	0.000	59.52	60.71	44.05
		0.200	57.14	61.90	44.05
		0.500	50.00	57.14	40.48
		0.700	42.86	55.95	29.76
	0.130	0.000	59.52	59.52	45.24
		0.200	57.14	61.90	45.24
		0.500	50.00	57.14	40.48
		0.700	42.86	54.76	29.76
	0.150	0.000	58.33	58.33	46.43
		0.200	57.14	61.90	46.43
		0.500	50.00	58.33	40.48
		0.700	44.05	54.76	28.57
0.003	0.100	0.000	59.52	58.33	44.05
		0.200	57.14	59.52	44.05
		0.500	51.19	57.14	39.29
		0.700	42.86	54.76	30.95
	0.110	0.000	59.52	58.33	44.05
		0.200	57.14	60.71	44.05
		0.500	51.19	57.14	39.29
		0.700	42.86	55.95	30.95
	0.120	0.000	59.52	60.71	44.05
		0.200	57.14	63.10	44.05
		0.500	50.00	57.14	39.29
		0.700	44.05	55.95	30.95
	0.130	0.000	59.52	58.33	45.24
		0.200	57.14	64.29	44.05
		0.500	50.00	57.14	40.48
		0.700	44.05	57.14	29.76
	0.150	0.000	59.52	59.52	45.24
		0.200	57.14	63.10	46.43
		0.500	50.00	58.33	40.48
		0.700	44.05	55.95	28.57
0.005	0.100	0.000	59.52	59.52	44.05
		0.200	57.14	59.52	44.05
		0.500	51.19	55.95	39.29
		0.700	46.43	55.95	30.95
	0.110	0.000	59.52	57.14	44.05
		0.200	57.14	59.52	44.05
		0.500	51.19	55.95	39.29
		0.700	45.24	54.76	30.95
	0.120	0.000	59.52	58.33	44.05
		0.200	57.14	60.71	44.05
		0.500	51.19	55.95	39.29
		0.700	45.24	54.76	30.95
0.005	0.130	0.000	59.52	58.33	45.24
		0.200	57.14	60.71	44.05
		0.500	51.19	55.95	39.29
		0.700	45.24	55.95	29.76
	0.150	0.000	60.71	60.71	45.24
		0.200	57.14	61.90	46.43
		0.500	50.00	57.14	40.48
		0.700	45.24	54.76	29.76
0.010	0.100	0.000	59.52	58.33	44.05
		0.200	57.14	58.33	44.05
		0.500	51.19	55.95	39.29
		0.700	46.43	57.14	32.14
	0.110	0.000	59.52	59.52	44.05
		0.200	57.14	60.71	44.05
		0.500	51.19	55.95	39.29
		0.700	46.43	57.14	30.95
	0.120	0.000	60.71	57.14	44.05
		0.200	57.14	58.33	44.05
		0.500	51.19	55.95	39.29
		0.700	46.43	54.76	30.95
	0.130	0.000	60.71	57.14	44.05
		0.200	57.14	60.71	45.24
		0.500	51.19	57.14	39.29
		0.700	46.43	57.14	30.95
	0.150	0.000	60.71	59.52	45.24
		0.200	57.14	61.90	45.24
		0.500	51.19	57.14	39.29
		0.700	45.24	55.95	30.95
0.020	0.100	0.000	60.71	57.14	44.05
		0.200	58.33	58.33	44.05
		0.500	51.19	57.14	40.48
		0.700	47.62	53.57	33.33
	0.110	0.000	60.71	58.33	44.05
		0.200	58.33	59.52	45.24
		0.500	51.19	57.14	40.48
		0.700	47.62	57.14	33.33
	0.120	0.000	60.71	58.33	44.05
		0.200	58.33	59.52	45.24
		0.500	51.19	59.52	40.48
		0.700	47.62	55.95	33.33
	0.130	0.000	60.71	59.52	44.05
		0.200	58.33	59.52	45.24
		0.500	51.19	58.33	40.48
		0.700	47.62	54.76	33.33
	0.150	0.000	60.71	58.33	45.24
		0.200	58.33	59.52	45.24
		0.500	51.19	57.14	40.48
		0.700	47.62	54.76	33.33

References

1. T Poisot, E Canard, D Mouillot, N Mouquet, D Gravel, The dissimilarity of species interaction networks. *Ecol. Lett.* **15**, 1353–1361 (2012).
2. PJ CaraDonna, et al., Interaction rewiring and the rapid turnover of plant–pollinator networks. *Ecol. Lett.* **20**, 385–394 (2017).
3. T Poisot, Dissimilarity of species interaction networks: quantifying the effect of turnover and rewiring. (2021).
4. T Petanidou, AS Kallimanis, J Tzanopoulos, SP Sgardelis, JD Pantis, Long-term observation of a pollination network: fluctuation in species and interactions, relative invariance of network structure and implications for estimates of specialization. *Ecol. letters* **11**, 564–75 (2008).
5. RP Rohr, S Saavedra, J Bascompte, On the structural stability of mutualistic systems. *Science* **345**, 1253497–1253497 (2014).
6. V Domínguez-Garcia, FP Molina, O Godoy, I Bartomeus, Interaction network structure explains species' temporal persistence in empirical plant–pollinator communities. *Nat. Ecol. & Evol.* **8**, 423–429 (2024).
7. S Jonhson, V Domínguez-García, MA Muñoz, Factors determining nestedness in complex networks. *PLoS ONE* **8**, e74025 (2013).
8. G Yan, ND Martinez, YY Liu, Degree heterogeneity and stability of ecological networks. *J. The Royal Soc. Interface* **14**, 20170189 (2017).
9. S Saavedra, RP Rohr, JM Olesen, J Bascompte, Nested species interactions promote feasibility over stability during the assembly of a pollinator community. *Ecol. Evol.* **6**, 997–1007 (2016).
